# Identification of common signature genes and pathways underlying the pathogenesis association between nonalcoholic fatty liver disease and heart failure

**DOI:** 10.3389/fimmu.2024.1424308

**Published:** 2024-09-16

**Authors:** Gerui Li, Zhengjie Lu, Ze Chen

**Affiliations:** ^1^ Department of Cardiology, Zhongnan Hospital of Wuhan University, Wuhan, China; ^2^ Department of Geriatrics, Zhongnan Hospital of Wuhan University, Wuhan, China; ^3^ Institute of Myocardial Injury and Repair, Wuhan University, Wuhan, China; ^4^ Division of Joint Surgery and Sports Medicine, Department of Orthopedic Surgery, Zhongnan Hospital of Wuhan University, Wuhan, China

**Keywords:** nonalcoholic fatty liver disease, heart failure, bioinformatics, biomarker, macrophage polarization

## Abstract

**Background:**

Non-alcoholic fatty liver disease (NAFLD) and heart failure (HF) are related conditions with an increasing incidence. However, the mechanism underlying their association remains unclear. This study aimed to explore the shared pathogenic mechanisms and common biomarkers of NAFLD and HF through bioinformatics analyses and experimental validation.

**Methods:**

NAFLD and HF-related transcriptome data were extracted from the Gene Expression Omnibus (GEO) database (GSE126848 and GSE26887). Differential analysis was performed to identify common differentially expressed genes (co-DEGs) between NAFLD and HF. Gene ontology (GO), Kyoto Encyclopedia of Genes and Genomes (KEGG), and gene set enrichment analysis (GSEA) were conducted to explore the functions and regulatory pathways of co-DEGs. Protein-protein interaction (PPI) network and support vector machine-recursive feature elimination (SVM-RFE) methods were used to screen common key DEGs. The diagnostic value of common key DEGs was assessed by receiver operating characteristic (ROC) curve and validated with external datasets (GSE89632 and GSE57345). Finally, the expression of biomarkers was validated in mouse models.

**Results:**

A total of 161 co-DEGs were screened out in NAFLD and HF patients. GO, KEGG, and GSEA analyses indicated that these co-DEGs were mainly enriched in immune-related pathways. PPI network revealed 14 key DEGs, and SVM-RFE model eventually identified two genes (*CD163* and *CCR1*) as common key DEGs for NAFLD and HF. Expression analysis revealed that the expression levels of *CD163* and *CCR1* were significantly down-regulated in HF and NAFLD patients. ROC curve analysis showed that *CD163* and *CCR1* had good diagnostic values for HF and NAFLD. Single-gene GSEA suggested that *CD163* and *CCR1* were mainly engaged in immune responses and inflammation. Experimental validation indicated unbalanced macrophage polarization in HF and NAFLD mouse models, and the expression of CD163 and CCR1 were significantly down-regulated.

**Conclusion:**

This study identified M2 polarization impairment characterized by decreased expression of CD163 and CCR1 as a common pathogenic pathway in NAFLD and HF. The downregulation of CD163 and CCR1 may reflect key pathological changes in the development and progression of NAFLD and HF, suggesting their potential as diagnostic and therapeutic targets.

## 1 Introduction

Heart failure (HF) is a significant health issue worldwide, with a high prevalence and substantial burden on individuals, healthcare systems, and society as a whole. It is associated with significant morbidity and mortality rates and is a leading cause of hospitalization (1). A 2017 Global Burden of Disease Study report estimated that approximately 64.34 million individuals worldwide suffer from HF (2). The prevalence of HF rises sharply with age. It has been estimated that approximately 10% of community-dwelling individuals aged ≥ 60 years have HF, with HF with preserved ejection fraction (HFpEF) being more prevalent than HF with reduced ejection fraction (HFrEF) (5% vs 3.5%) (3). Nonalcoholic fatty liver disease (NAFLD) has emerged as the most common chronic liver disease, affecting around 25% of adults worldwide (4). It encompasses a spectrum of liver conditions ranging from simple steatosis to nonalcoholic steatohepatitis (NASH), which is characterized by lobular inflammation and hepatocyte ballooning (with or without hepatic fibrosis) (5, 6). In some cases, NASH can progress to advanced liver fibrosis and cirrhosis and may even lead to hepatocellular carcinoma (7). The global prevalence of NAFLD is projected to significantly rise in the coming years, mirroring the escalating epidemics of obesity and type 2 diabetes (T2D) (8). Thus, how to deal with NAFLD and HF, two burdensome diseases worldwide, is a remarkable challenge.

Compelling evidence indicates that NAFLD is linked not just to increased liver-related complications but also to an elevated risk of extra-hepatic complications such as cardiovascular diseases (CVDs) (9–11). Of note, 25%-40% of individuals with NAFLD have CVDs, which stands as the primary cause of mortality in this patient group (12). NAFLD not only accelerates coronary artery disease, but also induces myocardial alterations (primarily cardiac remodeling and hypertrophy) and certain arrhythmias (mostly atrial fibrillation), conferring an increased risk of HF (13). A meta-analysis of 16 cross-sectional studies involving 32,000 subjects revealed that imaging-diagnosed NAFLD was associated with subclinical myocardial structural changes, such as increased left ventricular mass, alongside reduced early diastolic relaxation (e’) velocity, elevated left ventricular filling pressure, and enlarged left atrial volume (14). In addition, several studies have shown that the presence of NAFLD significantly raised the likelihood of developing new-onset HF, irrespective of whether T2D or other concurrent cardio-metabolic risk factors were present or not (12, 15). Notably, NAFLD has a stronger association with HFpEF than HFrEF. The prevalence of NAFLD is higher in patients with HFpEF than in those with HFrEF, reaching up to 50% (15, 16). Several studies also indicated that the presence of NAFLD is associated with a worse prognosis in patients with HF (17). The above epidemiological evidence indicates that NAFLD and HF are two closely associated entities. The strong association between NAFLD and HF warrants particular attention given its potential implications for screening and surveillance strategies in clinical practice.

However, there are still many unanswered questions regarding the relationship between NAFLD and HF. Particularly, whether there are shared pathophysiological pathways and common key molecules in the pathogenesis of NAFLD and HF are obscure but are of great importance to discover therapeutic approaches that benefit both diseases. Bioinformatics methods are extensively employed for mining transcriptome data to uncover the pathogenic mechanisms of diseases and to identify crucial molecular targets (18–20). This study aims to understand the common pathogenesis between NAFLD and HF and to unearth potential molecular targets of both diseases through bioinformatics analysis and experimental validation. In this study, human transcriptome data of NAFLD and healthy liver samples and HF and healthy heart samples were downloaded from the Gene Expression Omnibus (GEO) database. Employing a series of bioinformatics analysis methods such as protein-protein interaction (PPI) network analysis and support vector machine-recursive feature elimination (SVM-RFE), we systematically analyzed the common pathophysiological pathways between NAFLD and HF and mined key molecular biomarkers that may play a critical role in the pathogenesis of both diseases. Additionally, we validated the expression of these biomarkers in a NAFLD mouse model induced by a high-fat diet (HFD), a HFpEF mouse model induced by uninephrectomy surgery and d-aldosterone infusion, and a mouse model with both NAFLD and HF induced by long-term HFD. Overall, M2 polarization impairment characterized by decreased expression of CD163 and CCR1 was identified as a common pathogenic pathway in NAFLD and HF. The downregulation of CD163 and CCR1 may reflect key pathological changes in the development and progression of NAFLD and HF, suggesting their potential as diagnostic and therapeutic targets.

## 2 Materials and methods

### 2.1 Data source

To determine shared genetic interrelations between NAFLD and HF, four transcriptome datasets were obtained from the Gene Expression Omnibus (GEO, GSE126848, GSE89632, GSE26887, and GSE57345) database (http://www.ncbi.nlm.nih.gov/geo). The GSE126848 dataset contained the gene expression profiles of liver samples from 15 NAFLD patients and 14 healthy controls. The GSE89632 dataset contained the gene expression profiles of liver samples from 20 patients with NAFLD and 24 healthy controls. Meanwhile, the transcriptome data of heart samples from 12 HF patients and 5 non-HF controls were obtained from the GSE26887 dataset, and the transcriptome data of heart samples from 95 HF patients and 136 healthy controls were downloaded from the GSE57345 dataset. GSE126848 and GSE26887 datasets were used as test sets for differentially expressed genes (DEGs) analysis, whereas GSE89632 and GSE57345 datasets were used as validation sets.

### 2.2 Identification of differentially expressed genes in HF and NAFLD

DEGs between HF heart samples and healthy controls (GSE26887) and DEGs between NAFLD liver samples and corresponding controls (GSE126848) were identified utilizing the “limma” R package (version 3.46.0) (21). The selected criteria were set as *P*-value < 0.05 and |log_2_FC| > 0.5. Venn diagrams were used to identify co-DEGs in the two diseases.

### 2.3 Functional enrichment analyses for co-DEGs

To explore the potential mechanistic associations between NAFLD and HF, we conducted gene ontology (GO) and Kyoto Encyclopedia of Genes and Genomes (KEGG) pathway enrichment analyses of co-DEGs using the Database for Annotation, Visualization and Integrated Discovery (DAVID) bioinformatics resources (Version 6.8), available at https://david-d.ncifcrf.gov/. GO analysis was classified into three subgroups, including biological process (BP), molecular function (MF), and cellular component (CC). Functional groups with an Expression Analysis Systematic Explorer (EASE) score < 0.05 were included in this analysis. The EASE score, which is a modified Fisher Exact *P-*value in the DAVID system tailored for gene-enrichment analysis, signifies the degree of enrichment. Specifically, an EASE score *P*-value = 0 denotes a perfect enrichment. A *P*-value < 0.05 is indicative of gene enrichment in a specific annotation category. Moreover, gene set enrichment analysis (GSEA), a knowledge-based approach for interpreting genome-wide expression profiles, was employed on the co-DEGs using the “GSEA” R package (version 4.0.3) (22).

### 2.4 Protein-protein interaction network analysis and identification of common key DEGs

The PPI networks of co-DEGs were constructed utilizing the Search Tool for the Retrieval of Interacting Genes (STRING database; http://string-db.org/). This database predicted functional associations among proteins and protein-protein interactions, filtering for interaction scores exceeding 0.4 (23). The MCODE method in Cytoscape software was applied to analyze key modules in a PPI network (24). The degree, edge percolated component (EPC), maximum neighborhood component (MNC), and maximal clique centrality (MCC) algorithms in Cytoscape software were used to rank the co-DEGs in the PPI networks. The top 20 genes based on each algorithm were obtained, and the key DEGs were gained by taking the intersection set of the top 20 genes in the four algorithms. Subsequently, support vector machine-recursive feature elimination (SVM-RFE) method was applied to further sort these key DEGs in the GSE126848 and GSE26887 datasets separately, and the intersection of the two sorted gene sets was taken to obtain the common key DEGs.

### 2.5 Verification of the expression and diagnostic capacity of common key DEGs

The expression of common key DEGs was validated in the GSE126848, GSE26887, GSE89632 and GSE57345 datasets, respectively, using the Wilcox test method. In addition, we used the “pROC” R package (version 1.17.0.1) to plot receiver operating characteristic (ROC) curves of common key DEGs and assess their diagnostic capacity (25).

### 2.6 Single-gene GSEA

To explore the potential regulatory pathways and biological functions associated with the common key DEGs in the GSE126848 and GSE26887 datasets, the “GSEA” R package (version 4.0.3) was utilized to perform GSEA of each common key DEG (22). The adjusted *P* < 0.05 was considered as the significance threshold for GSEA.

### 2.7 Protein-ligand interaction and chemical-protein interaction analysis

To explore potential drug candidates, the STITCH (http://stitch.embl.de/) database was used to predict the pairs of interactions between common key DEGs and chemical drugs as well as related proteins. In addition, the GeneMANIA database (http://genemania.org/), a reliable online tool for discerning internal correlations in gene sets, was used to construct the regulatory network for the common key DEGs.

### 2.8 Animals and treatments

Forty eight male C57BL/6J mice, aged 8 weeks, were housed in standard conditions (ambient temperature: 23 ± 2°C; 12-hour light/dark cycle) with ad libitum access to water and standard laboratory chow. Following a one-week acclimation period, 24 mice were randomly assigned to either a normal chow (NC) group or a high-fat diet (HFD) group (n = 12 per group). The NC group received standard laboratory chow, while the HFD group was fed a high-fat diet (60% kcal from fat; D12492, Research Diets, New Brunswick, NJ, USA). After 14 weeks of dietary intervention, mice were anesthetized with 2% isoflurane and euthanized, and serum and liver samples were collected (26). Twelve mice were randomly assigned to either a control (CON) group or a HFpEF group following one week of acclimation (n = 6 per group). All 12 mice underwent uninephrectomy and received a continuous infusion of either saline (CON) or d-aldosterone (0.15mg/h) (HFpEF) for 4 weeks via osmotic mini-pumps (Alzet, Durect Corp., Cupertino, CA, United States) (27–29). Following the treatment period, mice were anesthetized with 2% isoflurane and euthanized, and their heart samples were weighed and collected. Another 12 mice were randomly assigned to either a normal chow (NC) group or a high-fat diet (HFD) group (n = 6 per group). The NC group received standard laboratory chow, while the HFD group was fed a high-fat diet (60% kcal from fat; D12492, Research Diets, New Brunswick, NJ, USA). After 28 weeks of dietary intervention, mice were anesthetized with 2% isoflurane and euthanized, and serum, liver, and heart samples were collected (30–33).

### 2.9 Histology, immunofluorescence, and biochemical detection

Formalin-fixed mouse liver tissues underwent processing, and 5 μm-thick paraffin sections were cut and stained with hematoxylin-eosin (H&E) and oil red O. The histological characteristics were assessed using the NAFLD activity score (NAS) (34). For mouse heart samples, hearts were isolated and preserved in a 10% KCl solution. Subsequently, they were fixed in 4% paraformaldehyde for 5 days, embedded in paraffin, and sliced into approximately 5 µm sections. These sections were then stained with wheat germ agglutinin (WGA) to determine cardiomyocyte size. Immunofluorescence detection involved incubating 10 µm-thick sections with primary antibodies against CD163 (#GB11340-1-100, Servicebio) and CD80 (#GB114055-100, Servicebio), followed by incubation with FITC-conjugated anti-rabbit whole IgG and Texas Red-conjugated anti-mouse whole IgG. Microscopic observation and photography were conducted using 200x magnification, capturing 20 random fields of view for each sample. Serum alanine transaminase (ALT) levels were assessed using an ADVIA 2400 Chemistry System analyzer (Siemens, Tarrytown, NY, USA) following the manufacturer’s instructions. Liver triglyceride (TG) contents were measured using a commercial kit (#290-63701; Wako, Tokyo, Japan) according to the manufacturer’s protocol. Serum soluble CD163 (sCD163) levels were detected using a commercial kit (EM1475; Finetest, Wuhan, China) following the manufacturer’s protocol.

### 2.10 Total RNA extraction and real-time quantitative PCR analysis

Total RNA was extracted using Trizol (No. 15596026, Thermo Fisher Scientific, Waltham, MA, USA), followed by reverse transcription into cDNA using the HiScript III RT SuperMix (No. R323-01, Vazyme Biotech, Nanjing, Jiangsu, China) according to the manufacturer’s protocol. Quantitative PCR (qPCR) was carried out using Cham Q ™ Universal SYBR^®^ qPCR Master Mix (No. Q712-02, Vazyme Biotech, Nanjing, Jiangsu, China) and the QuantStudio^®^ 5 Real-Time PCR System (Thermo Fisher Scientific, Waltham, MA, USA). The expression levels of target genes were determined using the 2^-ΔΔCt^ method and normalized to glyceraldehyde-3-phosphate dehydrogenase (*Gapdh*).

### 2.11 Statistical analyses

All analyses were conducted using R statistical software (Version 4.2.2). Quantitative data in the experimental validation analyses were presented as mean ± standard error of the mean (S.E.M.). The D’Agostino & Pearson normality test was employed to assess whether the data followed a parametric or non-parametric distribution. For parametric data comparing control and experimental groups, a two-tailed Student’s t-test was performed. In cases of datasets exhibiting skewed distribution, the Mann-Whitney U test was employed for group comparisons. A significance threshold of *P* < 0.05 (two-tailed) was considered statistically significant.

## 3 Results

### 3.1 Identification of co-DEGs between NAFLD and HF

**Figure 1:**
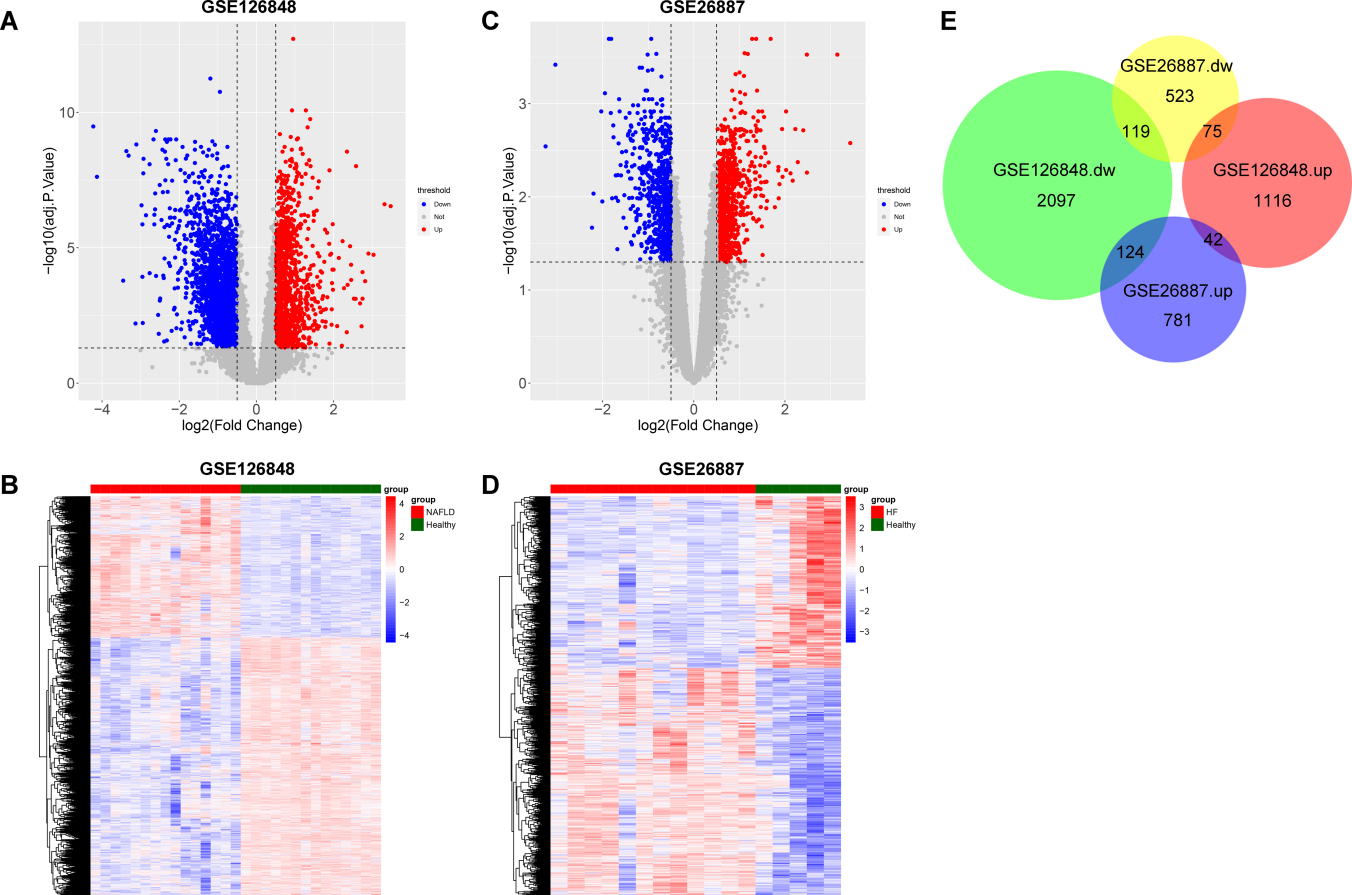
Identification of co-differentially expressed genes (DEGs) between non-alcoholic fatty liver disease (NAFLD) and heart failure (HF). **(A)** Volcano plot showing DEGs between the NAFLD group and the healthy control group in GSE126848, with upregulated genes indicated in red and downregulated genes in blue. **(B)** Heatmap showing the result of clustering analysis based on the expression of NAFLD-DEGs in GSE126848. **(C)** Volcano plot showing DEGs between the HF group and the healthy control group in GSE26887, with upregulated genes indicated in red and downregulated genes in blue. **(D)** Heatmap showing the result of clustering analysis based on the expression of HF-DEGs in GSE26887. **(E)** Proportional Venn diagram depicting the co-DEGs by overlapping NAFLD-DEGs and HF-DEGs.

To screen co**-**DEGs between NAFLD and HF, we first screened DEGs between NAFLD samples and healthy liver samples in the GSE126848 dataset. A total of 3623 DEGs, including 2340 down-regulated and 1283 up-regulated genes in NAFLD samples, were identified (**Figures 1A, B**). Meanwhile, DEGs were screened between HF samples and healthy heart samples in the GSE26887 dataset, and a total of 1664 DEGs were identified, including 717 down-regulated and 947 up-regulated genes in HF samples (**Figures 1C, D**). By intersecting the NAFLD- and HF-DEGs, we obtained 161 co-DEGs for subsequent analysis, among which 42 genes were up-regulated in both NAFLD and HF and 119 genes were down-regulated in both NAFLD and HF (**Figure 1E**).

### 3.2 Functional enrichment of co-DEGs

**Figure 2:**
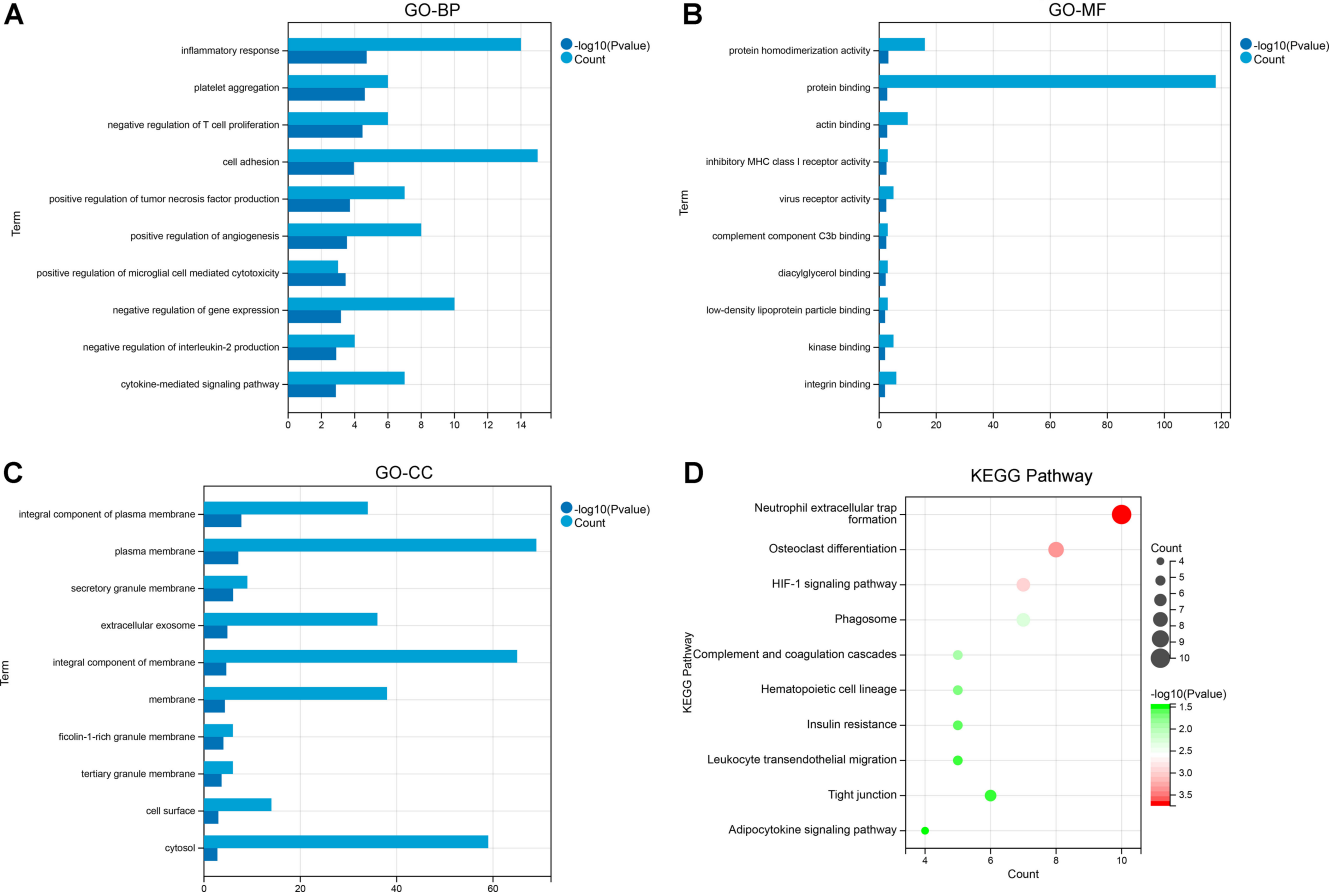
Gene ontology (GO) and Kyoto Encyclopaedia of Genes and Genomes (KEGG) functional enrichment analysis of co-differentially expressed genes (DEGs). **(A)** The enriched GO-biological process (BP) terms. **(B)** The enriched GO-molecular function (MF) terms. **(C)** The enriched GO-cellular component (CC) terms. **(D)** The enriched KEGG pathways.

To further explore the biological functions and signaling pathways involved in co-DEGs, we implemented GO functional analysis and KEGG pathway enrichment analysis. GO enrichment analysis showed that these co-DEGs were mainly involved in BP terms such as inflammatory response, platelet aggregation, negative regulation of T cell proliferation, cell adhesion, and positive regulation of tumor necrosis factor production (**Figure 2A**); MF terms such as protein homodimerization activity, protein binding, actin binding, inhibitory MHC class I receptor activity, and virus receptor activity (**Figure 2B**); and CC terms such as integral component of plasma membrane, plasma membrane, and secretory granule membrane (**Figure 2C**). In addition, the KEGG pathway analysis indicated that the co-DEGs were mainly enriched in pathways such as neutrophil extracellular trap formation, osteoclast differentiation, HIF-1 signaling pathway, phagosome, and complement and coagulation cascades (**Figure 2D**).

**Figure 3:**
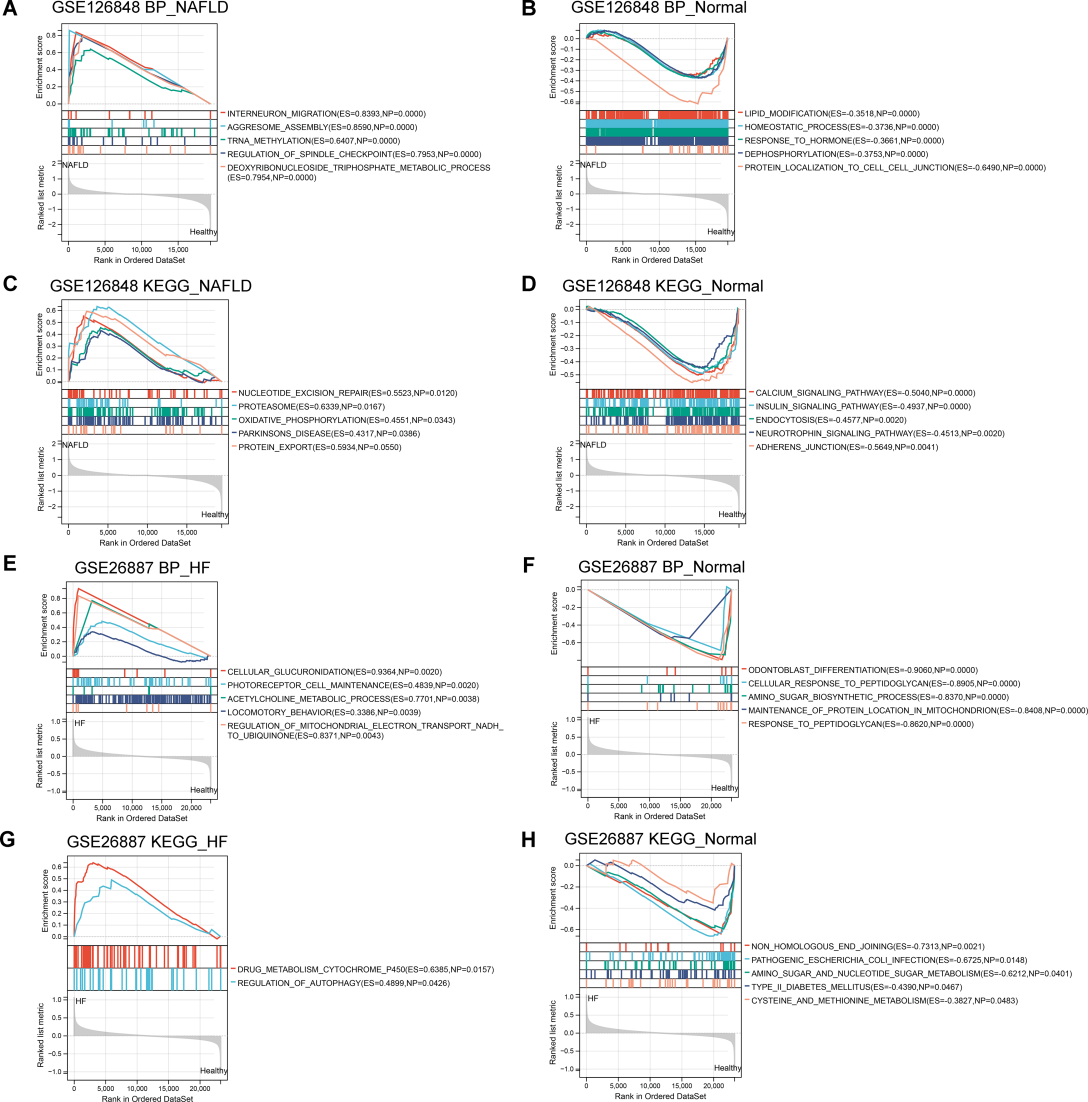
Gene Set Enrichment Analysis (GSEA) of co-differentially expressed genes (DEGs) in GSE126848 and GSE26887 datasets. **(A, B)** Biological processes found by GSEA in NAFLD **(A)** and normal **(B)** groups in GSE126848 dataset. **(C, D)** KEGG pathways enriched by GSEA in NAFLD **(C)** and normal **(D)** groups in GSE126848 dataset. **(E, F)** Biological processes found by GSEA in heart failure **(E)** and normal **(F)** groups in GSE26887 dataset. **(G, H)** KEGG pathways enriched by GSEA in heart failure **(G)** and normal **(H)** groups in GSE26887 dataset.

To further investigate the potential functions of these co-DEGs, we performed GSEA in the GSE126848 and GSE26887 datasets. In the GSE126848 dataset, GSEA results showed that biological processes such as aggresome assembly were enriched in the NAFLD group (**Figure 3A**), whereas biological processes such as lipid modifications were enriched in the normal sample group (**Figure 3B**). Meanwhile, KEGG pathways such as nucleotion excision repair and oxidative phosphorylation were enriched in the NAFLD group (**Figure 3C**), whereas KEGG pathways such as calcium signaling pathway and insulin signaling were enriched in the normal group (**Figure 3D**). In the GSE26887 dataset, GSEA results showed that biological processes such as cellular glucuronidation were enriched in the HF group (**Figure 3E**), whereas biological processes such as cellular response to peptidoglycan were enriched in the normal sample group (**Figure 3F**). Meanwhile, KEGG pathways such as drug metabolism cytochrome p450 and regulation of autophagy were enriched in the HF group (**Figure 3G**), whereas KEGG pathways such as pathogenic Escherichia coli infection and type II diabetes mellitus were enriched in the normal group (**Figure 3H**).

### 3.3 Identification of common key DEGs by PPI network analysis and a machine learning model

**Figure 4:**
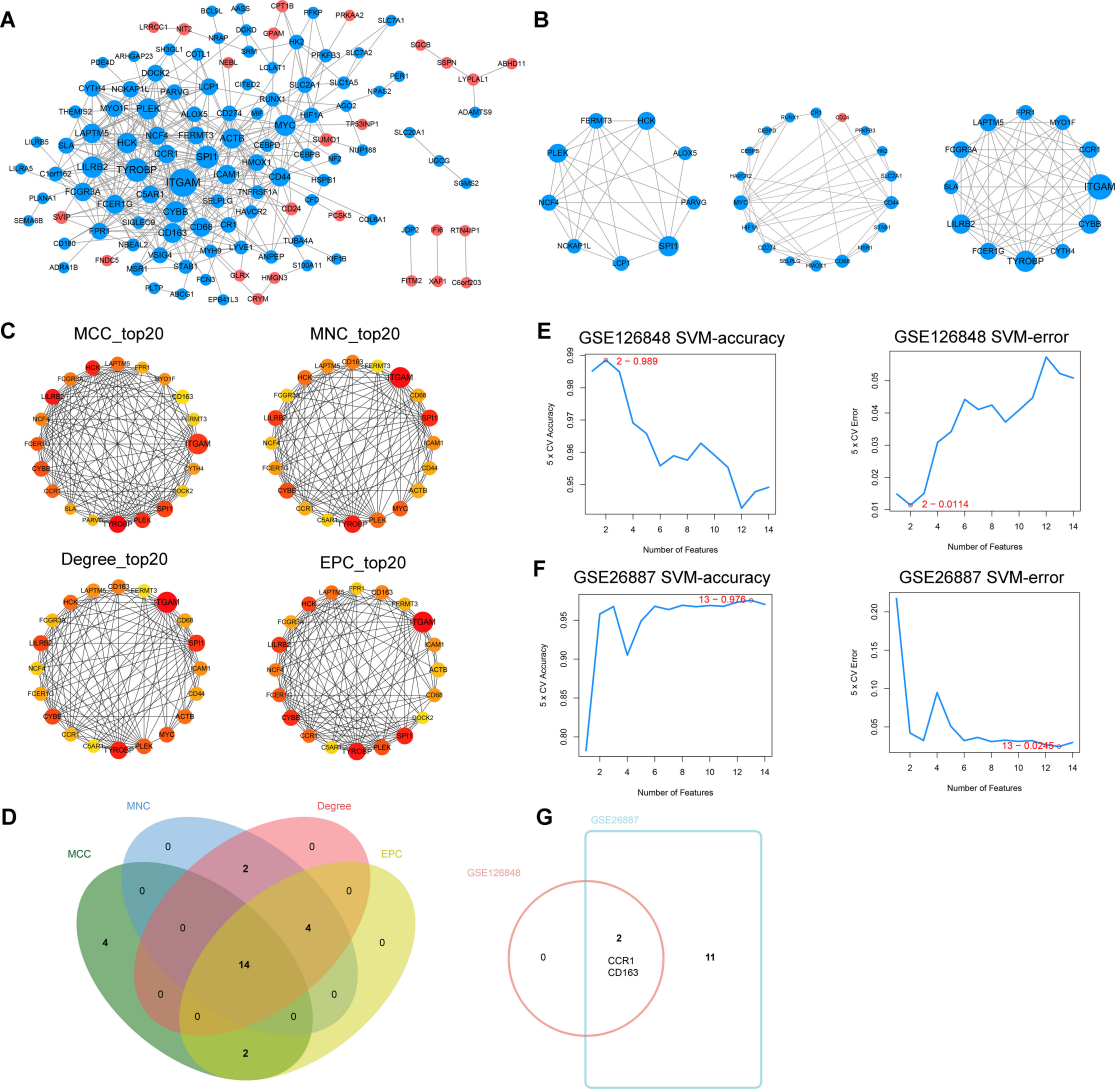
Identification of common key differentially expressed genes (DEGs). **(A)** Protein-protein interaction (PPI) network of co-DEGs. **(B)** The three significant modules in the PPI network found by MCODE plugin. **(C)** Top 20 genes based on the maximal clique centrality (MCC), maximum neighborhood component (MNC), Degree, and edge percolated component (EPC) algorithms, respectively. **(D)** Venn diagram showing the key DEGs obtained by taking the intersection set of the top 20 genes in the four algorithms. **(E)** Accuracy and error plots of SVM-RFE model in GSE126848 dataset. **(F)** Accuracy and error plots of SVM-RFE model in GSE26887 dataset. **(G)** Common key DEGs obtained by taking the intersection of the key DEGs in GSE126848 and GSE26887.

To explore the interactions among the 161 co-DEGs, the STRING website was applied to map the PPI network. We identified 435 interactions and 118 nodes from the PPI network of co-DEGs (**Figure 4A**) and obtained three significant modules using the MCODE plugin (**Figure 4B**). The MCC, MNC, Degree and EPC algorithms in Cytoscape software were used to rank the co-DEGs. The top 20 genes based on each algorithm were obtained (**Figure 4C**) and then 14 key DEGs (*TYROBP*, *LILRB2*, *HCK*, *PLEK*, *ITGAM*, *SPI1*, *CYBB*, *FCER1G*, *CCR1*, *LAPTM5*, *FCGR3A*, *NCF4*, *FERMT3*, and *CD163*) were gained by further taking the intersection set of the top 20 genes in each algorithm (**Figure 4D**). These key DEGs were further sorted by using the SVM-RFE method in the GSE126848 (**Figure 4E**) and GSE26887 (**Figure 4F**) datasets separately (**Table 1**), and two common key DEGs (*CCR1* and *CD163*) were finally obtained by taking the intersection of the two sorted gene sets (**Figure 4G**).

**Table 1:** Genes sorted by support vector machine-recursive feature elimination (SVM-RFE) method.

Feature Name	Feature ID	Average Rank
GSE126848		
CCR1	10	1.6
CD163	13	1.8
GSE26887		
CD163	2	2.0
LILRB2	10	3.2
ITGAM	8	3.6
CCR1	1	5.6
HCK	7	6.8
FERMT3	6	7.2
LAPTM5	9	7.6
FCER1G	4	7.8
TYROBP	14	9.2
CYBB	3	9.4
SPI1	13	10.2
FCGR3A	5	10.4
NCF4	11	10.8

### 3.4 Validation of the expression and diagnostic capacity of *CCR1* and *CD163*

**Figure 5:**
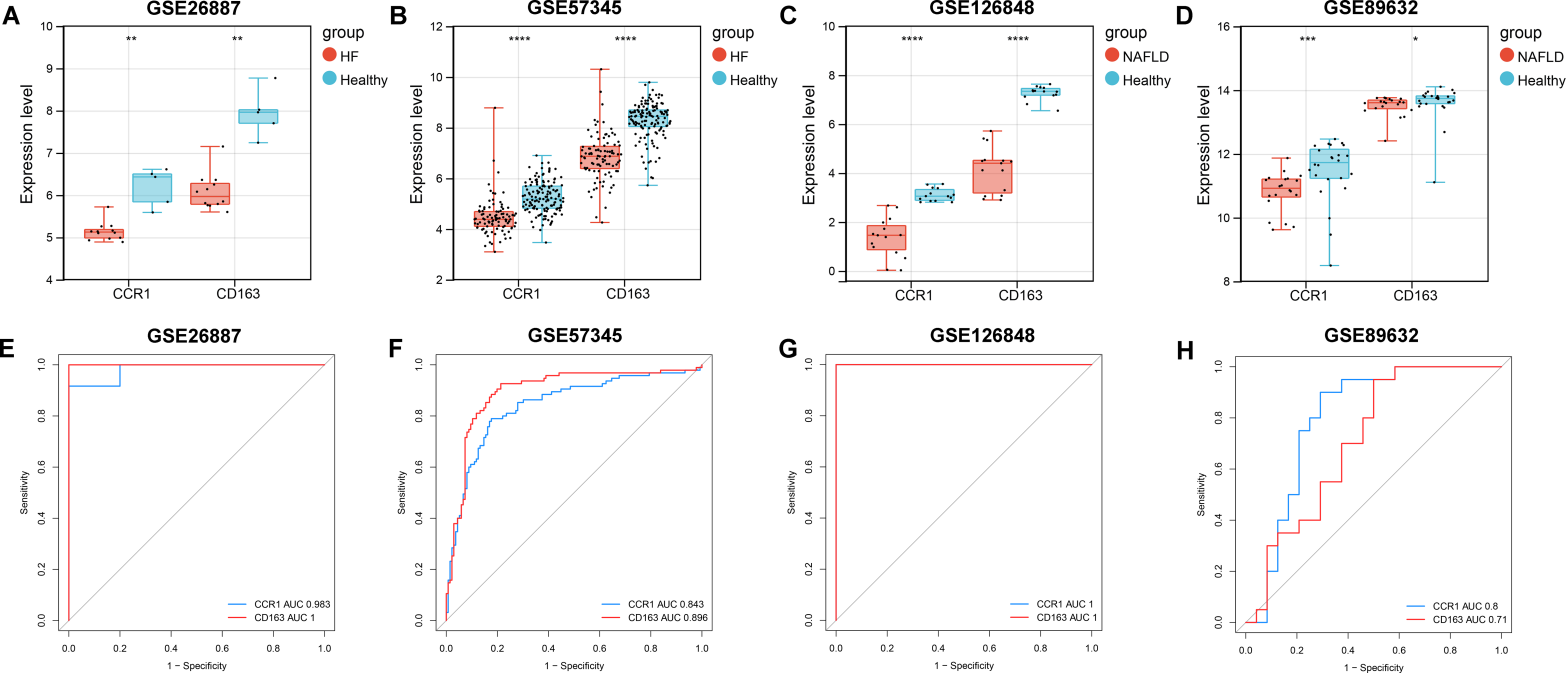
Validation and receiver operating characteristic (ROC) curve analyses of *CCR1* and *CD163* in heart failure (HF) and nonalcoholic fatty liver disease (NAFLD). **(A–D)** Expression of *CCR1* and *CD163* in GSE26887 **(A)**, GSE57345 **(B)**, GSE126848 **(C)**, and GSE89632 **(D)**, respectively. **(E–H)** ROC curve and area under the curve (AUC) of *CCR1* and *CD163* in GSE26887 **(E)**, GSE57345 **(F)**, GSE126848 **(G)**, and GSE89632 **(H)**, respectively. *P < 0.05, **P < 0.01, ***P < 0.001, ****P < 0.0001 vs. healthy controls.

To further validate the expression of *CCR1* and *CD163* in NALFD and HF and explore their diagnostic capacity, we performed validation and ROC curve analyses in HF (GSE26887 and GSE57345) and NAFLD (GSE126848 and GSE89632) datasets. Validation analyses showed that the expression of *CCR1* and *CD163* were consistently and significantly decreased in both HF datasets (GSE26887 and GSE57345) and in both NAFLD datasets (GSE126848 and GSE89632) (**Figures 5A–D**). ROC curve analyses indicated that the AUC values of *CCR1* and *CD163* were above 0.7 in all of the four datasets, indicating that *CCR1* and *CD163* had a good diagnostic capacity for HF and NAFLD (**Figures 5E–H**).

### 3.5 Singe-gene GSEA of *CCR1* and *CD163* in NAFLD and HF

**Figure 6:**
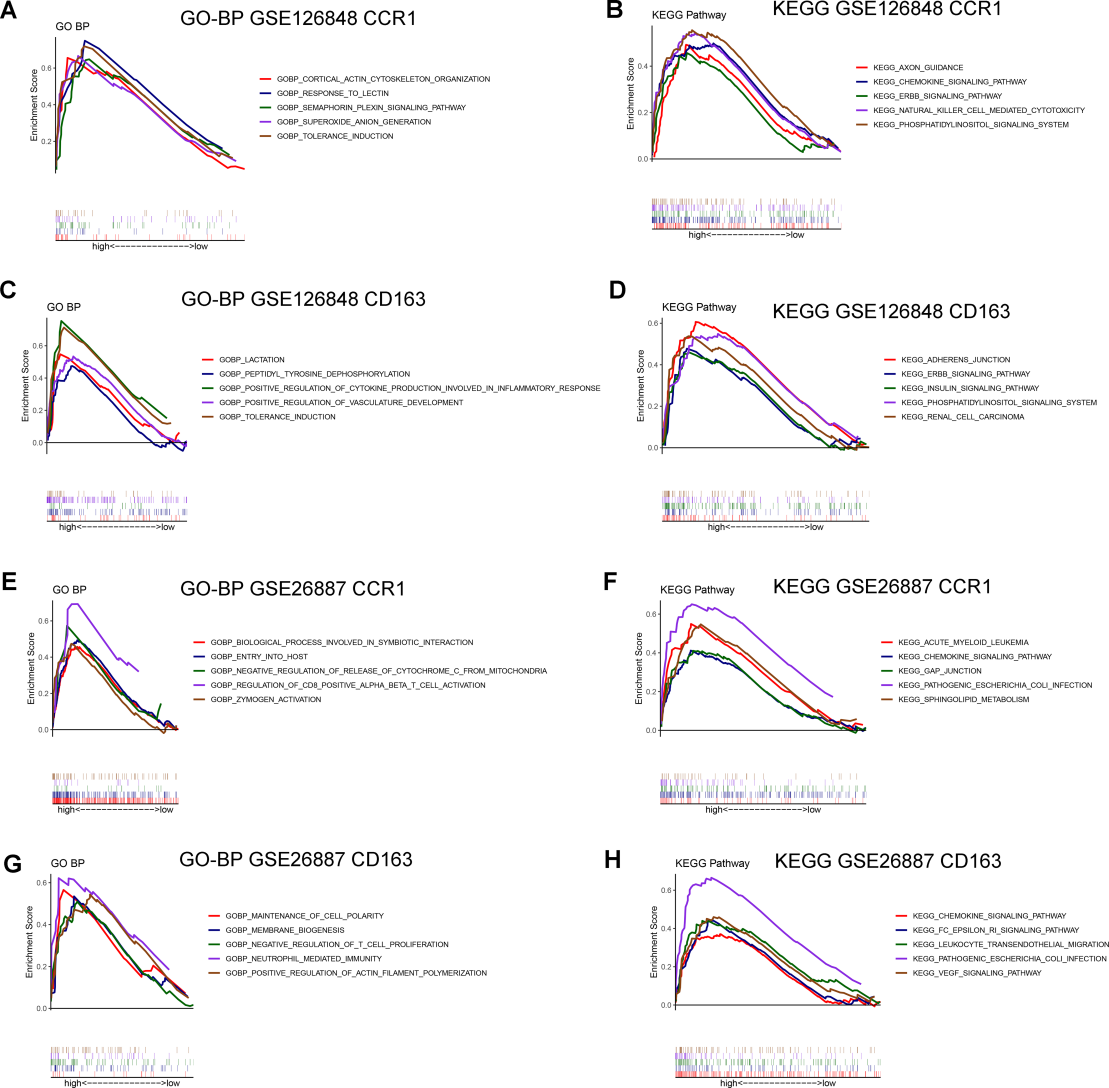
Gene Set Enrichment Analysis (GSEA) of *CCR1* and *CD163* in GSE126848 and GSE26887 datasets. **(A, B)** Biological processes **(A)** and KEGG pathways **(B)** found by singe-gene GSEA of *CCR1* in NAFLD dataset GSE126848. **(C, D)** Biological processes **(C)** and KEGG pathways **(D)** found by singe-gene GSEA of *CD163* in NAFLD dataset GSE126848. **(E, F)** Biological processes **(E)** and KEGG pathways **(F)** found by singe-gene GSEA of *CCR1* in HF dataset GSE26887. **(G, H)** Biological processes **(G)** and KEGG pathways **(H)** found by singe-gene GSEA of *CD163* in HF dataset GSE26887.

To explore the potential regulatory pathways and biological functions associated with *CCR1* and *CD163* in NAFLD (GSE126848) and HF (GSE26887), we performed singe-gene GSEA in the two datasets. In the GSE126848 dataset, *CCR1* was mainly involved in biological processes such as superoxide anion generation and response to lectin (**Figure 6A**) and KEGG pathways such as chemokine signaling pathway and natural killer cell mediated cytotoxicity (**Figure 6B**), whereas *CD163* was mainly involved in biological processes such as tolerance induction and positive regulation of cytokine production involved in inflammatory response (**Figure 6C**) and KEGG pathways such as adherens junction and insulin signaling pathway (**Figure 6D**). In the GSE26887 dataset, *CCR1* was mainly involved in biological processes such as negative regulation of release of cytochrome C from mitochondria and regulation of CD8 positive alpha beta T cell activation (**Figure 6E**) and KEGG pathways such as chemokine signaling pathway and pathogenic Escherichia coli infection (**Figure 6F**), whereas *CD163* was mainly involved in biological processes such as maintenance of cell polarity and neutrophil mediated immunity (**Figure 6G**) and KEGG pathways such as chemokine signaling pathway and leukocyte transendothelial migration (**Figure 6H**).

### 3.6 Interaction of CCR1 and CD163 with inflammatory genes and chemical drugs

**Figure 7:**
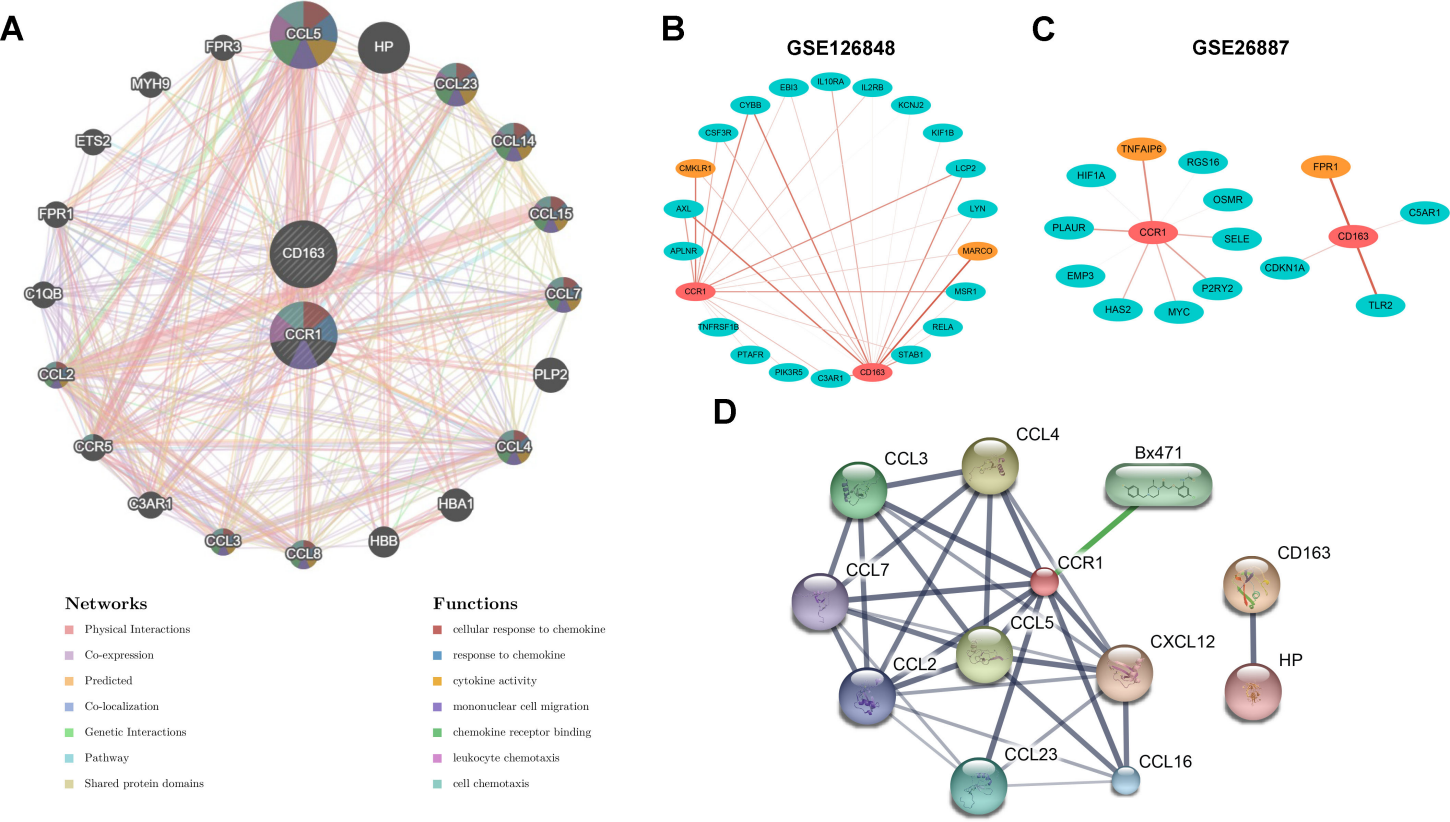
Interaction of CCR1 and CD163 with inflammatory genes and chemical drugs. **(A)** Regulatory network of *CCR1* and *CD163* and their co-expression genes constructed by the GeneMANIA database. **(B, C)** The correlation between *CCR1* and *CD163* with inflammation-related genes in the GSE126848 **(B)** and GSE26887 **(C)** datasets. **(D)** The interactions between CCR1 and CD163 with chemicals and proteins constructed by the STITCH database.

To further investigate the regulatory role of CCR1 and CD163, we constructed a regulatory network for CCR1 and CD163 using the GeneMANIA database (**Figure 7A**). Among the 20 targets of the network, 9 belonged to the CC ligand chemokine family (CCL5, CCL23, CCL14, CCL15, CCL7, CCL4, CCL8, CCL3, CCL2). Functional analyses of the network indicated these targets were involved in cellular response to chemokine, response to chemokine, cytokine activity, mononuclear cell migration, chemokine receptor binding, leukocyte chemotaxis, and cell chemotaxis. Next, to explore the correlation between *CCR1* and *CD163* with inflammation-related genes, we calculated the correlation between *CCR1* and *CD163* with inflammation-related genes in the GSE126848 and GSE26887 datasets, respectively. A total of 33 significant relationship pairs were obtained in the GSE126848 dataset, and *CCR1* and *CD163* had the strongest correlations with *CMKLR1* and *MARCO* (**Figure 7B**). In the GSE26887 dataset, a total of 14 significant pairs of relationships were obtained, and the strongest correlations were obtained between *CCR1* and *TNFAIP6* and between *CD163* and *FPR1* (**Figure 7C**). In addition, the STITCH database was applied to explore the interactions between CCR1 and CD163 with chemicals and proteins. As shown in **Figure 7D**, CCR1 interacted with the CCL family (CCL2, CCL3, CCL4, CCL7, CCL5, CCL16, CCL23) and Bx47, whereas CD163 showed a strong interaction with HP (**Supplementary Table 1**).

### 3.7 Validation of CCR1 and CD163 in a NAFLD mouse model

**Figure 8:**
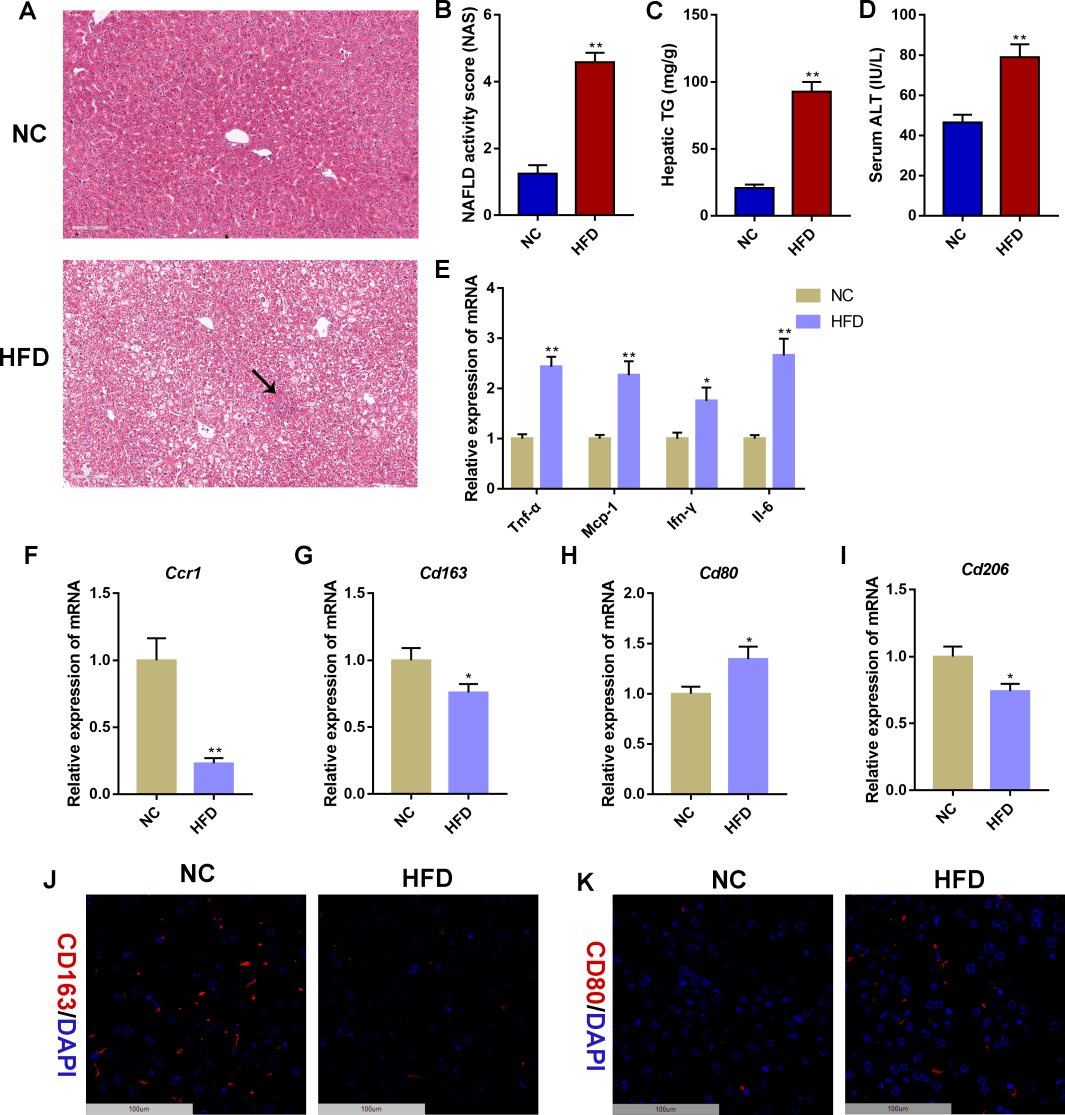
Validation of CCR1 and CD163 in a non-alcoholic fatty liver disease mouse model. **(A)** Hematoxylin&eosin (H&E) staining of liver tissues in mice with normal chow (10% of calorie from fat, NC) or high-fat diet (60% of calorie from fat, HFD) for 14 weeks. The black arrow indicates infiltrated immune cells. Scale bar = 100 μm. **(B)** NAFLD activity score (NAS) based on the H&E staining of liver tissues. **(C)** Hepatic triglyceride (TG) concentrations. **(D)** Serum alanine aminotransferase (ALT) levels. **(E)** Relative mRNA expression level of inflammatory marker genes in liver tissues. **(F–I)** Relative mRNA expression level of *Ccr1* **(F)**, *Cd163* **(G)**, *Cd80* **(H)**, and *Cd206* **(I)** in liver tissues. **(J)** Representative images under fluorescence microscopy showing CD163 staining (red) and nuclear staining (DIPA, blue) of liver tissues. Scale bar = 100 μm. **(K)** Representative images under fluorescence microscopy showing CD80 staining (red) and nuclear staining (DIPA, blue) of liver tissues. Scale bar = 100 μm. Mean ± S.E.M., *n* = 12. ^*^ *P<*0.05, ^**^ *P<*0.01 *vs.* the NC group.

To further verify the reliability of the in silico findings, we conducted validation in a NAFLD mouse model induced by 14 weeks of HFD feeding. Hepatic steatosis and sporadic inflammation were prominently observed in the HFD group, as demonstrated by H&E staining (**Figure 8A**). Compared to the NC group, the HFD group exhibited significantly elevated NAS, hepatic TG concentrations, and serum ALT levels (*P* < 0.01, **Figures 8B–D**). Additionally, mRNA expression levels of inflammatory markers (*Tnf-α*, *Mcp-1*, *Ifn-γ*, and *Il-6*) in liver tissues were significantly higher in the HFD group compared to the NC group (*P* < 0.05, *P* < 0.01, **Figure 8E**). Furthermore, mRNA expression validation indicated a significant decrease in the expression of hepatic *Ccr1* and *Cd163* in the HFD group compared to the NC group (*P* < 0.05, *P* < 0.01), consistent with human transcriptome data (**Figures 8F, G**). As CD163 and CCR1 are important players in macrophage polarization, we further detected the mRNA expression of M1 macrophage marker *Cd80* and M2 macrophage marker *Cd206* in liver tissues. Compared with the NC group, the mRNA expression of hepatic *Cd80* was significantly increased in the HFD group, whereas the mRNA expression of hepatic *Cd206* was significantly decreased in the HFD group (*P* < 0.05, **Figures 8H, I**). Moreover, immunofluorescence staining displayed decreased CD163-stained immunofluorescence (**Figure 8J**) and increased CD80-stained immunofluorescence (**Figure 8K**) in HFD livers than NC livers. The detection of serum sCD163 showed that HFD induced a reduction in the serum sCD163 level (*P* = 0.08, **Supplementary Figure S1A**). These results indicated that HFD feeding promotes macrophage polarization toward the M1 phenotype in the mouse liver.

### 3.8 Validation of CCR1 and CD163 in a HFpEF mouse model

**Figure 9:**
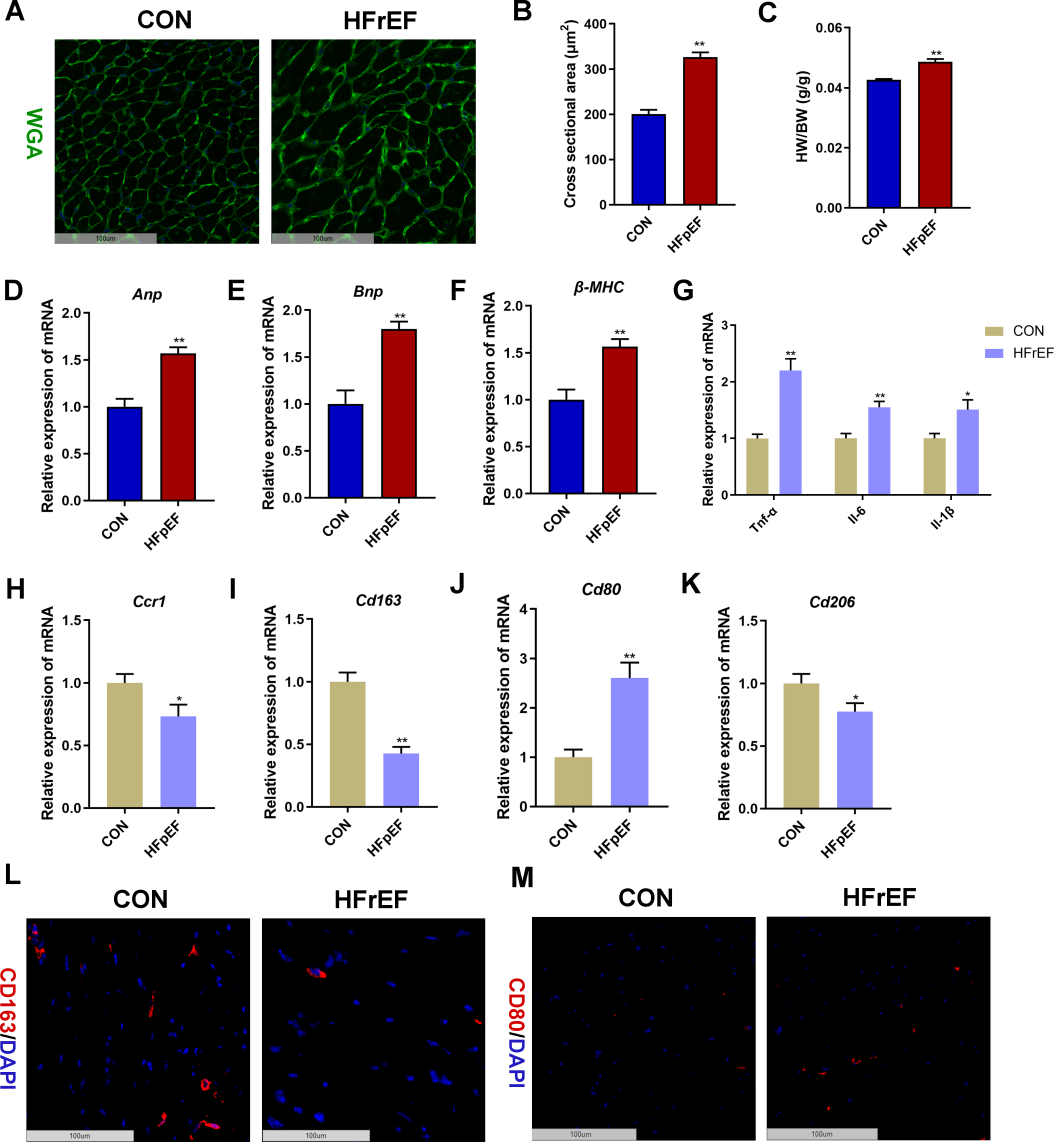
Validation of CCR1 and CD163 in a heart failure with reduced ejection fraction (HFpEF) mouse model. **(A)** Wheat germ agglutinin (WGA, green) staining of heart tissues in mice with saline (CON group) or uninephrectomy surgery followed by 0.15mg/h d-aldosterone (HFpEF group) treatment for 4 weeks. Scale bar = 100 μm. **(B)** Quantitative results of the left ventricular cross-sectional area based on the WGA staining of heart tissues. **(C)** Heart weight/body weight (HW/BW) ratio. **(D–F)** Relative mRNA expression level of left ventricular hypertrophy markers *Anp* **(D)**, *Bnp* **(E)**, and *β-MHC* **(F)** in heart tissues. **(G)** Relative mRNA expression level of inflammatory marker genes in heart tissues. **(H–K)** Relative mRNA expression level of *Ccr1* **(H)**, *Cd163* **(I)**, *Cd80* **(J)**, and *Cd206* **(K)** in heart tissues. **(L)** Representative images under fluorescence microscopy showing CD163 staining (red) and nuclear staining (DIPA, blue) of heart tissues. Scale bar = 100 μm. **(M)** Representative images under fluorescence microscopy showing CD80 staining (red) and nuclear staining (DIPA, blue) of heart tissues. Scale bar = 100 μm. Mean ± S.E.M., *n* = 6. ^*^ *P<*0.05, ^**^ *P<*0.01 *vs.* the control group.

We further validated the in silico results in a HFpEF mouse model induced by uninephrectomy and a continuous infusion of d-aldosterone for 4 weeks. WGA staining showed that the cross-sectional area of left ventricular cardiomyocytes in the HFpEF group was significantly larger than that in the control group (*P* < 0.01, **Figures 9A, B**). In addition, the heart weight-to-body weight (HW/BW) ratio (**Figure 9C**) and the mRNA expression level of left ventricular hypertrophy biomarkers (*Anp*, *Bnp*, and *β-MHC*, **Figures 9D–F**) and inflammatory markers (*Tnf-α*, *Il-6*, and *Il-1*, **Figure 9G**) in heart tissues were significantly higher in the HFpEF group than the control group (*P* < 0.05, *P* < 0.01). Furthermore, mRNA expression validation indicated a significant decrease in the expression of cardiac *Ccr1* and *Cd163* in the HFpEF group compared to the control group (*P* < 0.05, *P* < 0.01), consistent with human transcriptome data (**Figures 9H, I**). Compared with the control group, the mRNA expression of cardiac *Cd80* was significantly increased in the HFpEF group, whereas the mRNA expression of cardiac *Cd206* was significantly decreased in the HFpEF group (*P* < 0.05, *P* < 0.01, **Figures 9J, K**). Moreover, immunofluorescence staining displayed decreased CD163-stained immunofluorescence (**Figure 9L**) and increased CD80-stained immunofluorescence (**Figure 9M**) in HFpEF heart samples than control heart samples. These results indicated macrophage polarization toward the M1 phenotype in the HFpEF mouse heart.

### 3.9 Validation of CCR1 and CD163 in a mouse model with both NAFLD and HF

We further validated the in silico results in a mouse model with both NAFLD and HF induced by long-term HFD feeding, which is the most commonly used rodent model in the study of metabolism-related cardiomyopathy (30–33). H&E staining and oil red O staining of the liver tissues showed significant hepatic steatosis in the HFD group (**Supplementary Figures S2A, B**). Compared to the NC group, the HFD group exhibited significantly elevated NAS and hepatic TG concentrations (*P* < 0.01, **Supplementary Figures S2C, D**). Additionally, mRNA expression validation indicated a significant decrease in the expression of hepatic *Ccr1* and *Cd163* in the HFD group compared to the NC group (*P* < 0.05, **Supplementary Figures S2E, F**). WGA staining of the heart tissues showed that the cross-sectional area of left ventricular cardiomyocytes in the HFD group was significantly larger than that in the NC group (*P* < 0.01, **Supplementary Figures S2G, H**). In addition, the mRNA expression level of left ventricular hypertrophy biomarkers (*Anp* and *Bnp*) in heart tissues was significantly higher in the HFD group than in the NC group (*P* < 0.01, **Supplementary Figures S2I, J**). Furthermore, mRNA expression validation indicated a significant decrease in the expression of heart *Ccr1* and *Cd163* in the HFD group compared to the NC group (*P* < 0.05, **Supplementary Figures S2K, L**). These results indicated suppressed macrophage polarization toward the M2 phenotype in the liver and heart of mice with both NAFLD and HF induced by long-term over-nutrition.

## 4 Discussion

Accumulating epidemiological and clinical evidence supports a strong association between NAFLD and HF. However, the underlying pathophysiological mechanisms that link these two diseases remain unclear. In this study, we employed a series of bioinformatics approaches to analyze transcriptome data and validated the results in mouse models. We found that M2 polarization impairment characterized by decreased expression of CD163 and CCR1 was a common pathogenic pathway in NAFLD and HF. The downregulation of CD163 and CCR1 may reflect key pathological changes in the development and progression of NAFLD and HF, suggesting their potential as diagnostic and therapeutic targets.

The enrichment analysis indicated that co-DEGs of NAFLD and HF were mainly engaged in pathways related to immune responses. Indeed, the immune system plays a critical role in the pathogenesis of both NAFLD and HF (35, 36). NAFLD is now recognized as a systemic inflammatory disorder, with immune responses being central to its development and progression. The accumulation of excess lipids in the liver triggers metabolic stress and induces lipotoxicity, leading to the activation of inflammatory pathways and recruitment of immune cells (7). Resident liver cells, such as hepatocytes and Kupffer cells, release pro-inflammatory cytokines and chemokines in response to lipid overload. This triggers the recruitment of circulating immune cells, including monocytes and lymphocytes, to the liver, further amplifying the inflammatory responses (36). The role of immune responses in the pathogenesis of HF is also being increasingly recognized (35). In response to various insults such as myocardial infarction, hypertension, or chronic ischemia, the immune system becomes activated within the myocardium. This activation involves the recruitment of immune cells, including macrophages, neutrophils, and lymphocytes, to the heart tissue. These immune cells release pro-inflammatory cytokines contributing to myocardial inflammation, tissue injury, and adverse cardiac remodeling, which finally leads to HF (37, 38). Therefore, understanding the complex interplay between immune cells and signaling pathways in the pathogenesis of NAFLD and HF is essential for developing effective therapeutic strategies targeting inflammation and immune dysregulation in both organs.

A wealth of data has confirmed the significant contribution of macrophages to the development of NAFLD and HF (35, 36). The two major macrophage polarization states are classically activated (M1) and alternatively activated (M2) macrophages, each with distinct functions and cytokine profiles (39). In NAFLD, M1 macrophages contribute to the progression from simple steatosis to NASH by perpetuating liver inflammation and hepatocyte injury, whereas M2 macrophages have been shown to have protective effects by attenuating inflammation, promoting resolution of fibrosis, and enhancing lipid metabolism. The balance between M1 and M2 macrophages is crucial for the progression and resolution of NAFLD (40). Similarly, in both HFpEF and HFrEF, the imbalance in macrophage polarization, shifting between the pro-inflammatory M1 and anti-inflammatory M2 phenotypes, exacerbates inflammation and contributes to cardiac injury (27, 41, 42). In the present study, human transcriptome data analysis and animal experiments demonstrate that unbalanced macrophage polarization towards M1 polarization is a common pathophysiological process in NAFLD and HF. These results suggest that targeting macrophage polarization pathways represents a potential therapeutic strategy for both diseases, aiming to restore the balance between pro-inflammatory and anti-inflammatory macrophages and halt disease progression.

Two genes (*CD163* and *CCR1*) were screened out as common key DEGs of NAFLD and HF. Intriguingly, both genes have been reported as markers of macrophages and play an important role in macrophage polarization and function (43, 44). CD163 is a cell surface receptor predominantly expressed on macrophages and monocytes, particularly on the M2 subset. It is commonly used as a marker to identify M2-polarized macrophages (43). M2 macrophages produce anti-inflammatory cytokines which may attenuate hepatic inflammation and fibrogenesis in NAFLD (45, 46). Moreover, CD163 has garnered interest in HF research due to its potential roles in inflammation regulation, immune response modulation, and cardiovascular homeostasis (47, 48). Animal studies have shown that the expression of CD163 was downregulated in HF, and anti-HF treatment could reverse the expression of CD163 in the heart (27). Thus, given its multi-faceted roles, CD163 may serve as a promising diagnostic and therapeutic target for NAFLD and HF. Our ROC curve analyses indicated that *CD163* had a good diagnostic capacity for both diseases. Of note, recent clinical studies have shown that sCD163, released from activated macrophages, may serve as a biomarker in NAFLD and HF (48–51). Further research is needed to elucidate the precise mechanisms underlying CD163-mediated effects in NAFLD and HF and to validate its translational potential as a biomarker.

CCR1 is a chemokine receptor that has been implicated in various inflammatory and immune-mediated disorders. CCR1, along with its ligands, such as CCL3 and CCL5, plays a critical role in the recruitment and activation of immune cells, particularly macrophages and monocytes, at sites of inflammation (52). Although the role and mechanisms of CCR1 in macrophage polarization are still being elucidated, emerging studies suggest that CCR1 could influence macrophage polarization in the context of inflammation and immune responses, mostly by promoting M2 macrophage activation and function (44, 53, 54). For instance, CCR1 was found preferentially expressed on M2 macrophages compared with M1 macrophages, and M2 macrophages expressing high levels of CCR1 have the advantage of migration to inflammatory lesions, thus decreasing the local inflammation in healthy individuals (44). CCR1 is also reported as a M2 macrophage-related gene in chronic rhinosinusitis with nasal polyps (53). Moreover, the blockade of CCR1 inhibited protumor M2-like macrophage phenotype by decreasing CD206 and IL-10 expression and triggered a favorable anti-lymphoma activity (54). Our ROC curve analyses indicated that *CCR1* had a good diagnostic capacity for HF and NAFLD. However, the role of CCR1 in macrophage polarization in the context of NAFLD and HF and the translational potential of CCR1 as a diagnostic and therapeutic biomarker need to be further investigated.

It is important to note that several studies reported an increased CCR1 expression in human NASH liver tissues and elevated levels of serum sCD163 in NASH patients (55–57). The inconsistent results may result from the differences in the stages and severity of NAFLD between our study and others. In the present study, the gene expression profiles of human livers and HFD mouse models were at an early stage of NAFLD (i.e., the simple steatosis stage). To further investigate the expression of CCR1 and CD163 in the NASH stage, we applied a NASH model induced by 4 weeks of methionine/choline-deficient (MCD) diet feeding. In contrast to the HFD-induced NAFL model, the mRNA expression level of hepatic *Ccr1* and *Cd163* and serum level of sCD163 were significantly increased in MCD-induced NASH mice (**Supplementary Figures S1, S3**). Since NAFLD is an extremely complex clinical condition with a broad disease spectrum ranging from simple steatosis to steatohepatitis and eventually to advanced fibrosis and hepatocellular carcinoma, the expression and role of specific genes may vary among different stages of NAFLD (58–61). Furthermore, multiple macrophage populations co-exist at different stages in NAFLD livers, either facilitating or hindering disease progression, yet the intricate interplay among these populations and their communication with other cells in the liver environment remains inadequately elucidated (62, 63). The functions of CCR1 and CD163 as well as macrophage polarization in different stages of NAFLD and during the progression from one stage to the next require further investigation.

## 5 Limitations and future perspectives

There are several limitations in this study. First, this study aims to identify biomarkers and therapeutic targets for individuals with both diseases, however, the comorbidity information of NAFLD and HF is lacking in the gene expression profiling datasets. Thus, although this study validated the findings in a mouse model with both NAFLD and HF, further studies are needed to verify the expression of CD163 and CCR1 in clinical samples from patients with both diseases. Second, the molecular functions of CD163 and CCR1 were not validated in this study, thus the potential therapeutic benefits of manipulating the expression of CD163 and CCR1 in NAFLD and HF remain hypothetical. Further *in vivo* and *in vitro* studies with loss/gain of function experiments are imperative to elucidate the functions, underlying mechanisms, and translational potential of CD163 and CCR1 in NAFLD and HF. Moreover, the expression of CD163 and CCR1 in the peripheral blood of patients with NAFLD and HF needs to be verified in future studies to reveal their capacity for diagnosis and risk stratification.

## 6 Conclusions

In conclusion, this study identified that M2 polarization impairment characterized by decreased expression of CD163 and CCR1 is a common pathogenic pathway in NAFLD and HF. The downregulation of CD163 and CCR1 may reflect key pathological changes in the development and progression of NAFLD and HF, suggesting their potential as diagnostic and therapeutic targets. This study emphasizes the tight pathogenic interactions between NAFLD and HF and highlights the importance of targeting both organs of the same patient in further studies.

## Data Availability

The datasets presented in this study can be found in online repositories. The names of the repository/repositories and accession number(s) can be found in the article/**Supplementary Material**.

## References

[1] Heidenreich PA Bozkurt B Aguilar D Allen LA Byun JJ Colvin MM. Aha/Acc/Hfsa guideline for the management of heart failure: A report of the american college of cardiology/American heart association joint committee on clinical practice guidelines. Circulation. (2022) 145:e895–e1032. doi: 10.1161/CIR.0000000000001142 35363499

[2] Disease GBD Injury I Prevalence C. Global, regional, and national incidence, prevalence, and years lived with disability for 354 diseases and injuries for 195 countries and territories, 1990-2017: A systematic analysis for the global burden of disease study 2017. Lancet. (2018) 392:1789–858. doi: 10.1016/S0140-6736(18)32279-7 PMC6227754 30496104

[3] van Riet EE Hoes AW Wagenaar KP Limburg A Landman MA Rutten FH. Epidemiology of heart failure: the prevalence of heart failure and ventricular dysfunction in older adults over time. A Systematic Review. Eur J Heart Fail. (2016) 18:242–52. doi: 10.1002/ejhf.483 26727047

[4] Chalasani N Younossi Z Lavine JE Charlton M Cusi K Rinella M The diagnosis and management of nonalcoholic fatty liver disease: Practice guidance from the American Association for the Study of Liver Diseases. Hepatology. (2018) 67:328–57. doi: 10.1002/hep.29367 28714183

[5] Chen Z Yu Y Cai J Li H. Emerging molecular targets for treatment of nonalcoholic fatty liver disease. Trends Endocrinol Metab. (2019) 30:903–14. doi: 10.1016/j.tem.2019.08.006 31597607

[6] Chen Z Xia LP Shen L Xu D Guo Y Wang H. Glucocorticoids and intrauterine programming of nonalcoholic fatty liver disease. Metabolism. (2024) 150:155713. doi: 10.1016/j.metabol.2023.155713 37914025

[7] Chen Z Tian R She Z Cai J Li H. Role of oxidative stress in the pathogenesis of nonalcoholic fatty liver disease. Free Radic Biol Med. (2020) 152:116–41. doi: 10.1016/j.freeradbiomed.2020.02.025 32156524

[8] Younossi Z Tacke F Arrese M Chander Sharma B Mostafa I Bugianesi E. Global perspectives on nonalcoholic fatty liver disease and nonalcoholic steatohepatitis. Hepatology. (2019) 69:2672–82. doi: 10.1002/hep.30251 30179269

[9] Chen Z Liu J Zhou F Li H Zhang XJ She ZG. Nonalcoholic fatty liver disease: an emerging driver of cardiac arrhythmia. Circ Res. (2021) 128:1747–65. doi: 10.1161/CIRCRESAHA.121.319059 34043417

[10] Li G Peng Y Chen Z Li H Liu D Ye X. Bidirectional association between hypertension and nafld: A systematic review and meta-analysis of observational studies. Int J Endocrinol. (2022) 2022:8463640. doi: 10.1155/2022/8463640 35371259 PMC8970889

[11] Zhao YC Zhao GJ Chen Z She ZG Cai J Li H. Nonalcoholic fatty liver disease: an emerging driver of hypertension. Hypertension. (2020) 75:275–84. doi: 10.1161/HYPERTENSIONAHA.119.13419 31865799

[12] Zhou J Bai L Zhang XJ Li H Cai J. Nonalcoholic fatty liver disease and cardiac remodeling risk: pathophysiological mechanisms and clinical implications. Hepatology. (2021) 74:2839–47. doi: 10.1002/hep.32072 34309877

[13] Cai J Zhang XJ Ji YX Zhang P She ZG Li H. Nonalcoholic fatty liver disease pandemic fuels the upsurge in cardiovascular diseases. Circ Res. (2020) 126:679–704. doi: 10.1161/CIRCRESAHA.119.316337 32105577

[14] Borges-Canha M Neves JS Libanio D Von-Hafe M Vale C Araujo-Martins M. Association between nonalcoholic fatty liver disease and cardiac function and structure-a meta-analysis. Endocrine. (2019) 66:467–76. doi: 10.1007/s12020-019-02070-0 31482382

[15] Mantovani A Byrne CD Benfari G Bonapace S Simon TG Targher G. Risk of heart failure in patients with nonalcoholic fatty liver disease: jacc review topic of the week. J Am Coll Cardiol. (2022) 79:180–91. doi: 10.1016/j.jacc.2021.11.007 35027111

[16] Miller A McNamara J Hummel SL Konerman MC Tincopa MA. Prevalence and staging of non-alcoholic fatty liver disease among patients with heart failure with preserved ejection fraction. Sci Rep. (2020) 10:12440. doi: 10.1038/s41598-020-69013-y 32709942 PMC7381602

[17] Qiu M Li J Hao S Zheng H Zhang X Zhu H. Non-alcoholic fatty liver disease is associated with a worse prognosis in patients with heart failure: A pool analysis. Front Endocrinol (Lausanne). (2023) 14:1167608. doi: 10.3389/fendo.2023.1167608 37152967 PMC10157242

[18] Li G Li H Chen Z. Identification of ribosomal protein family as immune-cell-related biomarkers of nafld by bioinformatics and experimental analyses. Front Endocrinol (Lausanne). (2023) 14:1161269. doi: 10.3389/fendo.2023.1161269 37274336 PMC10235545

[19] Peng C Zhang Y Lang X Zhang Y. Role of mitochondrial metabolic disorder and immune infiltration in diabetic cardiomyopathy: new insights from bioinformatics analysis. J Transl Med. (2023) 21:66. doi: 10.1186/s12967-023-03928-8 36726122 PMC9893675

[20] Zhu E Shu X Xu Z Peng Y Xiang Y Liu Y. Screening of immune-related secretory proteins linking chronic kidney disease with calcific aortic valve disease based on comprehensive bioinformatics analysis and machine learning. J Transl Med. (2023) 21:359. doi: 10.1186/s12967-023-04171-x 37264340 PMC10234004

[21] Ritchie ME Phipson B Wu D Hu Y Law CW Shi W. Limma powers differential expression analyses for rna-sequencing and microarray studies. Nucleic Acids Res. (2015) 43:e47. doi: 10.1093/nar/gkv007 25605792 PMC4402510

[22] Suarez-Farinas M Lowes MA Zaba LC Krueger JG. Evaluation of the psoriasis transcriptome across different studies by gene set enrichment analysis (Gsea). PloS One. (2010) 5:e10247. doi: 10.1371/journal.pone.0010247 20422035 PMC2857878

[23] Szklarczyk D Franceschini A Wyder S Forslund K Heller D Huerta-Cepas J. String V10: protein-protein interaction networks, integrated over the tree of life. Nucleic Acids Res. (2015) 43:D447–52. doi: 10.1093/nar/gku1003 PMC4383874 25352553

[24] Shannon P Markiel A Ozier O Baliga NS Wang JT Ramage D. Cytoscape: A software environment for integrated models of biomolecular interaction networks. Genome Res. (2003) 13:2498–504. doi: 10.1101/gr.1239303 PMC403769 14597658

[25] Robin X Turck N Hainard A Tiberti N Lisacek F Sanchez JC. Proc: an open-source package for R and S+ to analyze and compare roc curves. BMC Bioinf. (2011) 12:77. doi: 10.1186/1471-2105-12-77 PMC3068975 21414208

[26] Lee MR Yang HJ Park KI Ma JY. Lycopus lucidus turcz. Ex benth. Attenuates free fatty acid-induced steatosis in hepg2 cells and non-alcoholic fatty liver disease in high-fat diet-induced obese mice. Phytomedicine. (2019) 55:14–22. doi: 10.1016/j.phymed.2018.07.008 30668424

[27] Zhang L Chen J Yan L He Q Xie H Chen M. Resveratrol ameliorates cardiac remodeling in a murine model of heart failure with preserved ejection fraction. Front Pharmacol. (2021) 12:646240. doi: 10.3389/fphar.2021.646240 34177571 PMC8225267

[28] Shuai W Kong B Yang H Fu H Huang H. Loss of myeloid differentiation protein 1 promotes atrial fibrillation in heart failure with preserved ejection fraction. ESC Heart Fail. (2020) 7:626–38. doi: 10.1002/ehf2.12620 PMC7160510 31994333

[29] Zhang XL Wang TY Chen Z Wang HW Yin Y Wang L. Hmgb1-promoted neutrophil extracellular traps contribute to cardiac diastolic dysfunction in mice. J Am Heart Assoc. (2022) 11:e023800. doi: 10.1161/JAHA.121.023800 35156391 PMC9245819

[30] Shao D Kolwicz SC Jr Wang P Roe ND Villet O Nishi K. Increasing fatty acid oxidation prevents high-fat diet-induced cardiomyopathy through regulating parkin-mediated mitophagy. Circulation. (2020) 142:983–97. doi: 10.1161/CIRCULATIONAHA.119.043319 PMC7484440 32597196

[31] Tong M Saito T Zhai P Oka SI Mizushima W Nakamura M. Alternative mitophagy protects the heart against obesity-associated cardiomyopathy. Circ Res. (2021) 129:1105–21. doi: 10.1161/CIRCRESAHA.121.319377 34724805

[32] Zhu M Peng L Huo S Peng D Gou J Shi W. STAT3 signaling promotes cardiac injury by upregulating NCOA4-mediated ferritinophagy and ferroptosis in high-fat-diet fed mice. Free Radic Biol Med. (2023) 201:111–25. doi: 10.1016/j.freeradbiomed.2023.03.003 36940731

[33] Yang B Zhao Y Luo W Zhu W Jin L Wang M. Macrophage DCLK1 promotes obesity-induced cardiomyopathy via activating RIP2/TAK1 signaling pathway. Cell Death Dis. (2023) 14:419. doi: 10.1038/s41419-023-05960-4 37443105 PMC10345119

[34] Santhekadur PK Kumar DP Sanyal AJ. Preclinical models of non-alcoholic fatty liver disease. J Hepatol. (2018) 68:230–7. doi: 10.1016/j.jhep.2017.10.031 PMC5775040 29128391

[35] Zhang Y Bauersachs J Langer HF. Immune mechanisms in heart failure. Eur J Heart Fail. (2017) 19:1379–89. doi: 10.1002/ejhf.942 28891154

[36] Huby T Gautier EL. Immune cell-mediated features of non-alcoholic steatohepatitis. Nat Rev Immunol. (2022) 22:429–43. doi: 10.1038/s41577-021-00639-3 PMC8570243 34741169

[37] Chen Z Jin ZX Cai J Li R Deng KQ Ji YX. Energy substrate metabolism and oxidative stress in metabolic cardiomyopathy. J Mol Med (Berl). (2022) 100:1721–39. doi: 10.1007/s00109-022-02269-1 36396746

[38] Martini E Kunderfranco P Peano C Carullo P Cremonesi M Schorn T. Single-cell sequencing of mouse heart immune infiltrate in pressure overload-driven heart failure reveals extent of immune activation. Circulation. (2019) 140:2089–107. doi: 10.1161/CIRCULATIONAHA.119.041694 31661975

[39] Wang C Ma C Gong L Guo Y Fu K Zhang Y. Macrophage polarization and its role in liver disease. Front Immunol. (2021) 12:803037. doi: 10.3389/fimmu.2021.803037 34970275 PMC8712501

[40] Yoshii D Nakagawa T Komohara Y Kawaguchi H Yamada S Tanimoto A. Phenotypic changes in macrophage activation in a model of nonalcoholic fatty liver disease using microminipigs. J Atheroscler Thromb. (2021) 28:844–51. doi: 10.5551/jat.57703 PMC8326174 33012740

[41] Glezeva N Voon V Watson C Horgan S McDonald K Ledwidge M. Exaggerated inflammation and monocytosis associate with diastolic dysfunction in heart failure with preserved ejection fraction: evidence of M2 macrophage activation in disease pathogenesis. J Card Fail. (2015) 21:167–77. doi: 10.1016/j.cardfail.2014.11.004 25459685

[42] Mouton AJ Li X Hall ME Hall JE. Obesity, hypertension, and cardiac dysfunction: novel roles of immunometabolism in macrophage activation and inflammation. Circ Res. (2020) 126:789–806. doi: 10.1161/CIRCRESAHA.119.312321 32163341 PMC7255054

[43] Etzerodt A Moestrup SK. Cd163 and inflammation: biological, diagnostic, and therapeutic aspects. Antioxid Redox Signal. (2013) 18:2352–63. doi: 10.1089/ars.2012.4834 PMC3638564 22900885

[44] Nakano H Kirino Y Takeno M Higashitani K Nagai H Yoshimi R. Gwas-identified ccr1 and il10 loci contribute to M1 macrophage-predominant inflammation in behcet's disease. Arthritis Res Ther. (2018) 20:124. doi: 10.1186/s13075-018-1613-0 29895319 PMC5998575

[45] Ni Y Zhuge F Ni L Nagata N Yamashita T Mukaida N. Cx3cl1/cx3cr1 interaction protects against lipotoxicity-induced nonalcoholic steatohepatitis by regulating macrophage migration and M1/M2 status. Metabolism. (2022) 136:155272. doi: 10.1016/j.metabol.2022.155272 35914622

[46] Barreby E Chen P Aouadi M. Macrophage functional diversity in nafld - more than inflammation. Nat Rev Endocrinol. (2022) 18:461–72. doi: 10.1038/s41574-022-00675-6 35534573

[47] Chen C Peng H Zeng Y Dong G. Cd14, cd163, and ccr1 are involved in heart and blood communication in ischemic cardiac diseases. J Int Med Res. (2020) 48:300060520951649. doi: 10.1177/0300060520951649 32967511 PMC7521061

[48] Ptaszynska-Kopczynska K Marcinkiewicz-Siemion M Lisowska A Waszkiewicz E Witkowski M Jasiewicz M. Alterations of soluble tweak and cd163 concentrations in patients with chronic heart failure. Cytokine. (2016) 80:7–12. doi: 10.1016/j.cyto.2016.02.005 26916171

[49] Pourrajab B Naderi N Janani L Hajahmadi M Mofid V Dehnad A. The impact of probiotic yogurt versus ordinary yogurt on serum stweak, scd163, adma, lcat and bun in patients with chronic heart failure: A randomized, triple-blind, controlled trial. J Sci Food Agric. (2022) 102:6024–35. doi: 10.1002/jsfa.11955 35460085

[50] Rosso C Kazankov K Younes R Esmaili S Marietti M Sacco M. Crosstalk between adipose tissue insulin resistance and liver macrophages in non-alcoholic fatty liver disease. J Hepatol. (2019) 71:1012–21. doi: 10.1016/j.jhep.2019.06.031 31301321

[51] Kazankov K Moller HJ Lange A Birkebaek NH Holland-Fischer P Solvig J. The macrophage activation marker scd163 is associated with changes in nafld and metabolic profile during lifestyle intervention in obese children. Pediatr Obes. (2015) 10:226–33. doi: 10.1111/ijpo.252 25073966

[52] Barnes PJ. Chemokine receptor ccr1: new target for asthma therapy. Trends Pharmacol Sci. (2022) 43:539–41. doi: 10.1016/j.tips.2022.02.009 35246315

[53] Zhu Y Sun X Tan S Luo C Zhou J Zhang S. M2 macrophage-related gene signature in chronic rhinosinusitis with nasal polyps. Front Immunol. (2022) 13:1047930. doi: 10.3389/fimmu.2022.1047930 36466903 PMC9712459

[54] Le K Sun J Ghaemmaghami J Smith MR Ip WKE Phillips T. Blockade of ccr1 induces a phenotypic shift in macrophages and triggers a favorable antilymphoma activity. Blood Adv. (2023) 7:3952–67. doi: 10.1182/bloodadvances.2022008722 PMC10410136 36630565

[55] Li H Pan T Gao L Ding R Yu Y Ma M. Chemokine receptor CCR1 regulates macrophage activation through mTORC1 signaling in nonalcoholic steatohepatitis. Metabolism. (2024) 151:155758. doi: 10.1016/j.metabol.2023.155758 38070823

[56] Ragab HM El Maksoud NA Amin MA Elaziz WA. Performance of serum CD163 as a marker of fibrosis in patients with NAFLD. Diabetes Metab Syndr. (2021) 15:87–92. doi: 10.1016/j.dsx.2020.11.023 33310266

[57] Kawanaka M Nishino K Kawada M Ishii K Tanikawa T Katsumata R. Soluble CD163 is a predictor of fibrosis and hepatocellular carcinoma development in nonalcoholic steatohepatitis. BMC Gastroenterol. (2023) 23:143. doi: 10.1186/s12876-023-02786-4 37165352 PMC10173513

[58] Govaere O Cockell S Tiniakos D Queen R Younes R Vacca M. Transcriptomic profiling across the nonalcoholic fatty liver disease spectrum reveals gene signatures for steatohepatitis and fibrosis. Sci Transl Med. (2020) 12:eaba4448. doi: 10.1126/scitranslmed.aba4448 33268509

[59] Alshawsh MA Alsalahi A Alshehade SA Saghir SAM Ahmeda AF Al Zarzour RH. A comparison of the gene expression profiles of non-alcoholic fatty liver disease between animal models of a high-fat diet and methionine-choline-deficient diet. Molecules. (2022) 27:858. doi: 10.3390/molecules27030858 35164140 PMC8839835

[60] Suppli MP Rigbolt KTG Veidal SS Heebøll S Eriksen PL Demant M. Hepatic transcriptome signatures in patients with varying degrees of nonalcoholic fatty liver disease compared with healthy normal-weight individuals. Am J Physiol Gastrointest Liver Physiol. (2019) 316:G462–72. doi: 10.1152/ajpgi.00358.2018 30653341

[61] Hoang SA Oseini A Feaver RE Cole BK Asgharpour A Vincent R. Gene expression predicts histological severity and reveals distinct molecular profiles of nonalcoholic fatty liver disease. Sci Rep. (2019) 9:12541. doi: 10.1038/s41598-019-48746-5 31467298 PMC6715650

[62] Tacke F. Targeting hepatic macrophages to treat liver diseases. J Hepatol. (2017) 66:1300–1312. doi: 10.1016/j.jhep.2017.02.026 28267621

[63] Vonderlin J Chavakis T Sieweke M Tacke F. The multifaceted roles of macrophages in NAFLD pathogenesis. Cell Mol Gastroenterol Hepatol. (2023) 15:1311–24. doi: 10.1016/j.jcmgh.2023.03.002 PMC10148157 36907380

