# Supplementary material for: Identification of common signature genes and pathways underlying the pathogenesis association between nonalcoholic fatty liver disease and heart failure
Source: Front Immunol. 2024 Sep 16;15:1424308. doi: 10.3389/fimmu.2024.1424308 (PMC11439677; doi:10.3389/fimmu.2024.1424308)
Supplement: Supplementary file 1 [file DataSheet1.docx]

Supplementary Material


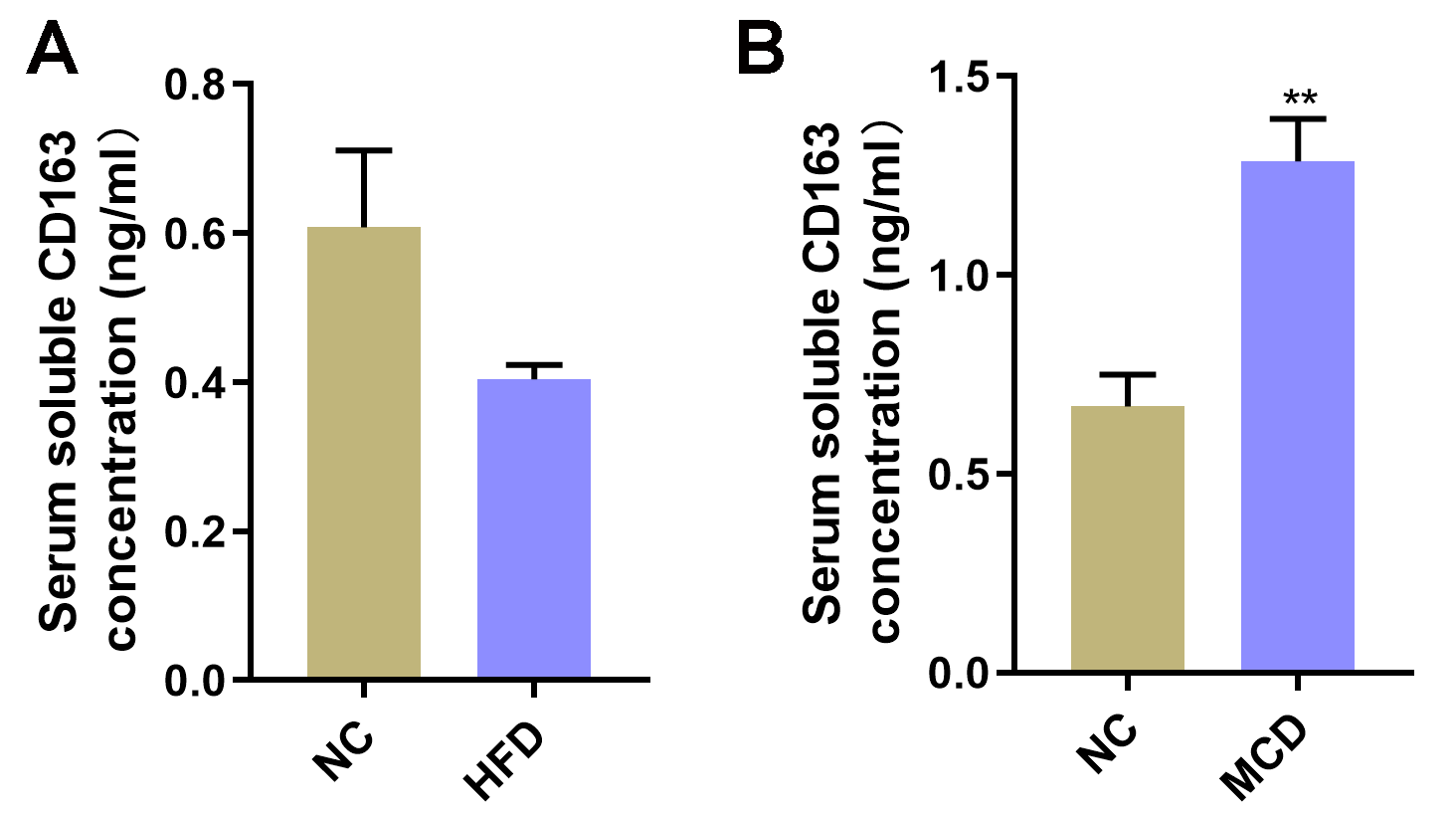


**Figure S1. Detection of serum soluble CD163 in non-alcoholic fatty liver disease mouse models.** (A) Serum soluble CD163 concentrations in mice with normal chow (10 % of calorie from fat, NC) or high-fat diet (60 % of calorie from fat, HFD) for 14 weeks, *n* = 12. (B) Serum soluble CD163 concentrations in mice with normal chow (NC) or methionine/choline-deficient (MCD) diet for 4 weeks, *n* = 6. Mean ± S.E.M. ^**^*P<*0.01 *vs.* the NC group.


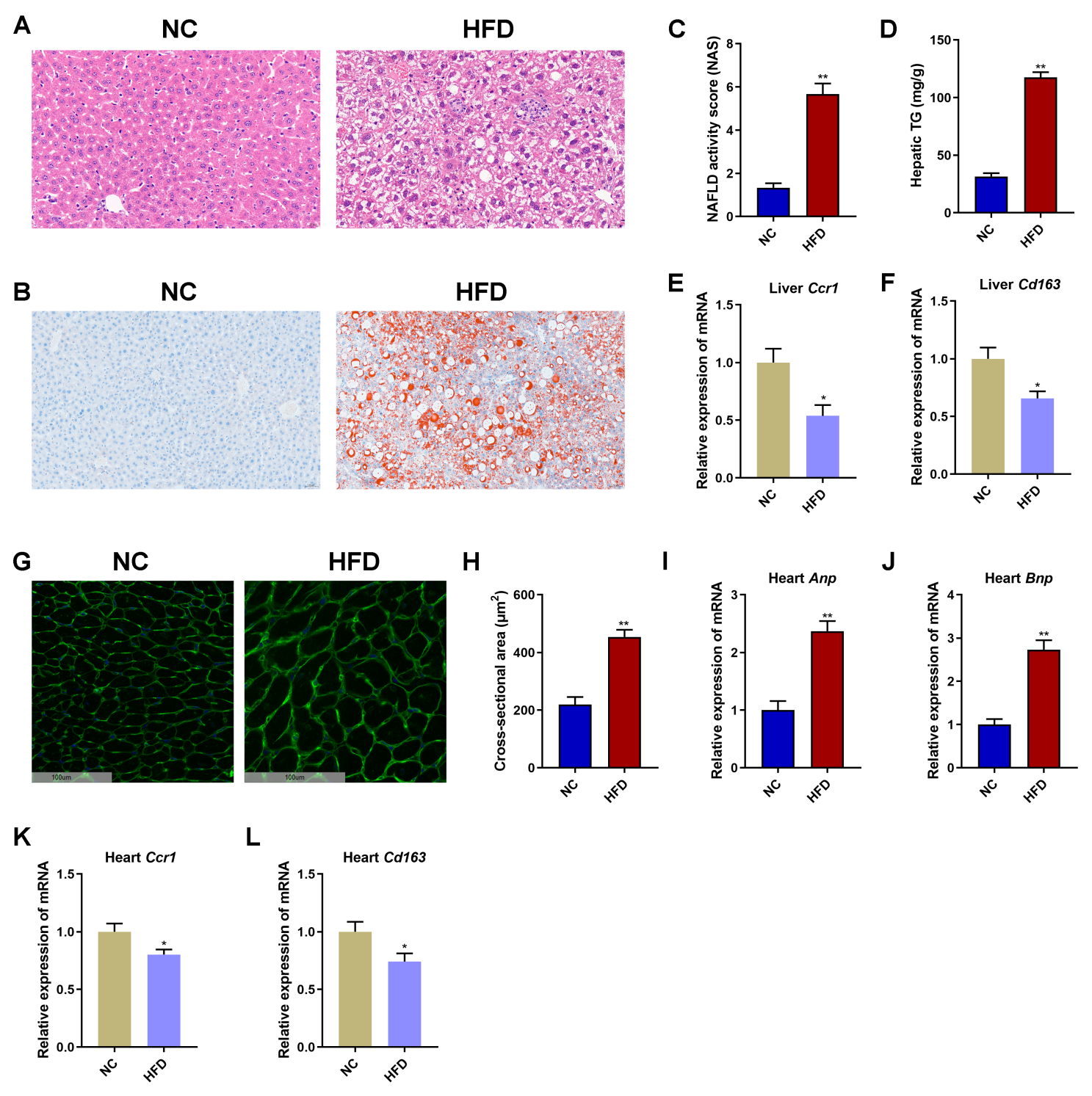


**Figure S2. Validation of CCR1 and CD163 in a mouse model with both non-alcoholic fatty liver disease and heart failure.** (A-B) Hematoxylin&eosin (H&E) staining (A) and oil red O staining (B) of liver tissues in mice with the normal chow (10 % of calorie from fat, NC) or high-fat diet (60 % of calorie from fat, HFD) for 28 weeks. Scale bar = 20 μm for H&E staining. Scale bar = 50 μm for oil red O staining. (C) NAFLD activity score (NAS) based on the H&E staining of liver tissues. (D) Hepatic triglyceride (TG) concentrations. (E-F) Relative mRNA expression level of *Ccr1* (E) and *Cd163* (F) in liver tissues. (G) Wheat germ agglutinin (WGA, green) staining of mouse heart tissues from the NC group and the HFD group. Scale bar = 100 μm. (H) Quantitative results of the left ventricular cross-sectional area based on the WGA staining of heart tissues. (I-J) Relative mRNA expression level of left ventricular hypertrophy markers *Anp* (I) and *Bnp* (J) in heart tissues. (K-L) Relative mRNA expression level of heart *Ccr1* (K) and *Cd163* (L) in heart tissues. Mean ± S.E.M., *n* = 6. ^*^*P<*0.05, ^**^*P<*0.01 *vs.* the NC group.

Twelve male C57BL/6J mice, aged 8 weeks, were housed in standard conditions (ambient temperature: 23 ± 2 °C; 12-hour light/dark cycle) with *ad libitum* access to water and standard laboratory chow. Following a one-week acclimation period, all mice were randomly assigned to either a normal chow (NC) group or a methionine/choline-deficient (MCD) diet group (n = 6 per group), and received standard laboratory chow or MCD diet (A02082002B, Research Diets), respectively. After 4 weeks of dietary intervention, mice were anesthetized with 2% isoflurane and euthanized. Serum and liver samples were collected for subsequent pathological staining, biochemical examination, and gene expression detection.

H&E staining of liver tissues showed marked hepatic steatosis and inflammation in the MCD group **(Figure S3A)**. Increased hepatic lipid deposition in the MCD group was further evidenced by oil red O staining **(Figure S3A)**. Sirius red staining demonstrated a significantly increased positive area in the MCD group compared with the NC group **(***P* < 0.01, **Figure S3A-B)**. Compared to the NC group, the MCD group exhibited significantly elevated serum ALT concentrations (*P* < 0.01, **Figure S3C**). Additionally, mRNA expression detection indicated a significant increase in the expression of hepatic *Ccr1* and *Cd163* in the MCD group compared to the NC group (*P* < 0.05, *P* < 0.01, **Figure S3D-E)**. The detection of serum sCD163 showed that the MCD diet induced a significant increase in the serum sCD163 level (*P* < 0.01, **Figure S1B**).


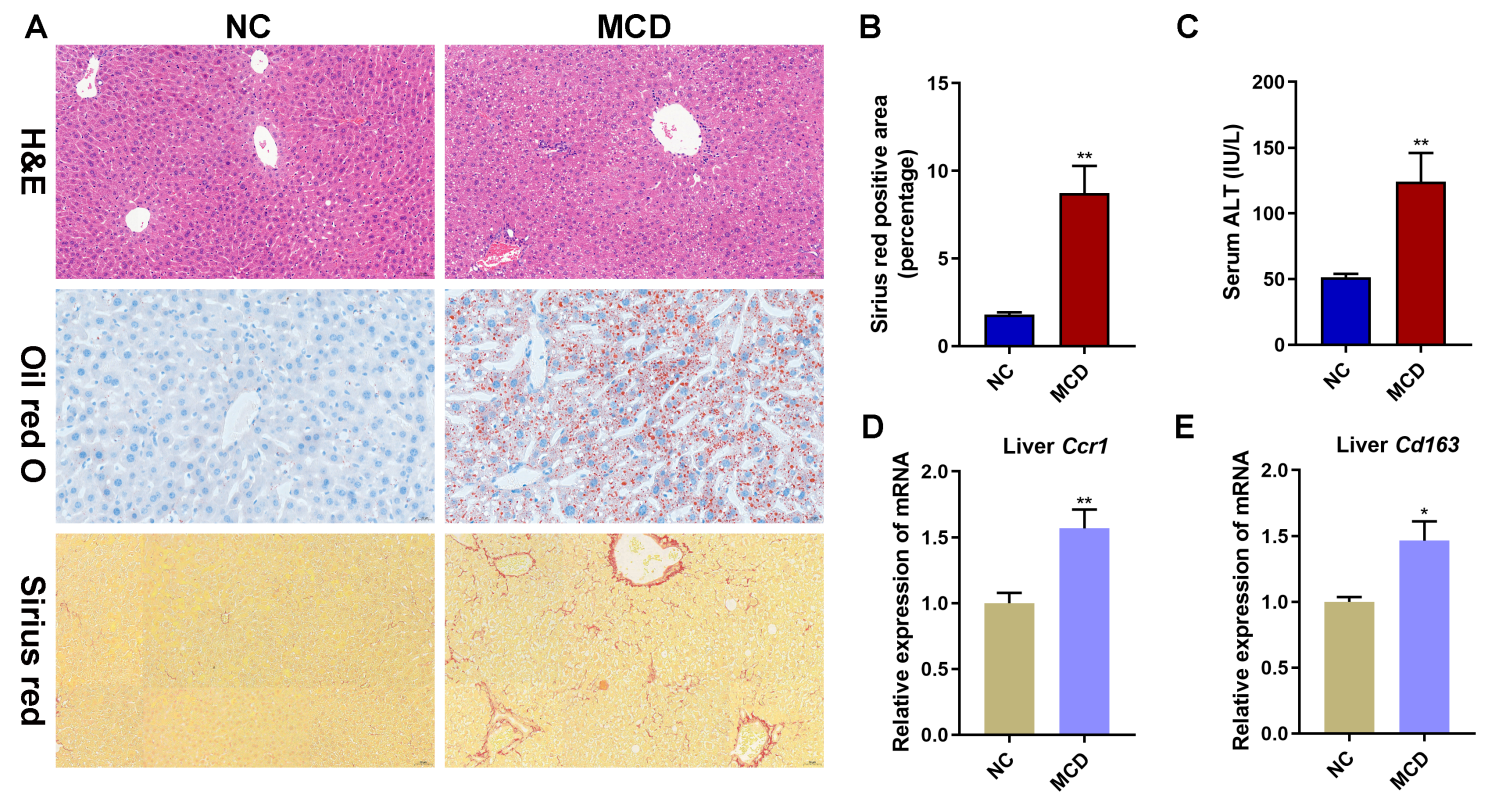


**Figure S3. Detection of CCR1 and CD163 expression in a non-alcoholic steatohepatitis mouse model.** (A-C) Hematoxylin&eosin (H&E, A), oil red O (B), and sirius red (C) staining of liver tissues in mice with normal chow (NC) or methionine/choline-deficient (MCD) diet for 4 weeks. Scale bar = 50 μm for H&E and sirius red staining. Scale bar = 20 μm for oil red O staining. (B) Sirius red positive area of liver tissues. (C) Serum alanine aminotransferase (ALT) levels. (D-E) Relative mRNA expression level of *Ccr1* (D) and *Cd163* (E) in liver tissues. Mean ± S.E.M., *n* = 6. ^*^*P<*0.05, ^**^*P<*0.01 *vs.* the NC group.

**Supplementary Table 1. The interactions between CCR1 and CD163 with chemicals and proteins predicted by the STITCH database.**

| **Node1** | **Node2** | **Node1 ID** | **Node2 ID** | **Experimentally determined interaction** | **Database annotated** | **Automated textmining** | **Combined score** |
| --- | --- | --- | --- | --- | --- | --- | --- |
| CCR1 | CCL4 | 9606.ENSP00000296140 | 9606.ENSP00000250151 | 0.807 | 0.8 | 0.989 | 0.999 |
| CCR1 | CCL5 | 9606.ENSP00000296140 | 9606.ENSP00000293272 | 0.961 | 0.9 | 0.757 | 0.998 |
| CCR1 | CCL3 | 9606.ENSP00000296140 | 9606.ENSP00000225245 | 0.807 | 0.8 | 0.949 | 0.997 |
| CCL5 | CCL2 | 9606.ENSP00000293272 | 9606.ENSP00000225831 | 0.566 | 0.9 | 0.909 | 0.995 |
| CCR1 | Bx471 | 9606.ENSP00000296140 | -1.CID100512282 | 0.923 | 0.8 | 0.37 | 0.989 |
| CCR1 | CCL16 | 9606.ENSP00000296140 | 9606.ENSP00000293275 | 0.802 | 0.9 | 0.472 | 0.988 |
| CCR1 | CCL23 | 9606.ENSP00000296140 | 9606.ENSP00000293280 | 0.812 | 0.8 | 0.725 | 0.988 |
| CCL7 | CCR1 | 9606.ENSP00000367832 | 9606.ENSP00000296140 | 0.807 | 0.8 | 0.7 | 0.987 |
| CCR1 | CCL2 | 9606.ENSP00000296140 | 9606.ENSP00000225831 | 0.812 | 0.8 | 0.705 | 0.987 |
| CD163 | HP | 9606.ENSP00000352071 | 9606.ENSP00000348170 | 0.576 | 0.9 | 0.682 | 0.985 |
| CXCL12 | CCR1 | 9606.ENSP00000379140 | 9606.ENSP00000296140 | 0 | 0.9 | 0.79 | 0.978 |
| CCL4 | CCL3 | 9606.ENSP00000250151 | 9606.ENSP00000225245 | 0.576 | 0.9 | 0.947 | 0.977 |
| CXCL12 | CCL5 | 9606.ENSP00000379140 | 9606.ENSP00000293272 | 0 | 0.9 | 0.72 | 0.97 |
| CXCL12 | CCL16 | 9606.ENSP00000379140 | 9606.ENSP00000293275 | 0 | 0.9 | 0.264 | 0.923 |
| CCL2 | CCL3 | 9606.ENSP00000225831 | 9606.ENSP00000225245 | 0 | 0.9 | 0.9 | 0.916 |
| CCL4 | CCL2 | 9606.ENSP00000250151 | 9606.ENSP00000225831 | 0 | 0.9 | 0.817 | 0.915 |
| CCL7 | CCL4 | 9606.ENSP00000367832 | 9606.ENSP00000250151 | 0 | 0.9 | 0.685 | 0.914 |
| CCL5 | CCL4 | 9606.ENSP00000293272 | 9606.ENSP00000250151 | 0 | 0.9 | 0.875 | 0.913 |
| CCL7 | CCL2 | 9606.ENSP00000367832 | 9606.ENSP00000225831 | 0 | 0.9 | 0.755 | 0.913 |
| CCL7 | CCL5 | 9606.ENSP00000367832 | 9606.ENSP00000293272 | 0 | 0.9 | 0.744 | 0.912 |
| CCL7 | CCL3 | 9606.ENSP00000367832 | 9606.ENSP00000225245 | 0 | 0.9 | 0.722 | 0.912 |
| CCL16 | CCL5 | 9606.ENSP00000293275 | 9606.ENSP00000293272 | 0 | 0.9 | 0.662 | 0.909 |
| CCL5 | CCL3 | 9606.ENSP00000293272 | 9606.ENSP00000225245 | 0 | 0.9 | 0.911 | 0.908 |
| CXCL12 | CCL4 | 9606.ENSP00000379140 | 9606.ENSP00000250151 | 0 | 0 | 0.752 | 0.752 |
| CXCL12 | CCL23 | 9606.ENSP00000379140 | 9606.ENSP00000293280 | 0 | 0 | 0.658 | 0.658 |
| CXCL12 | CCL3 | 9606.ENSP00000379140 | 9606.ENSP00000225245 | 0 | 0 | 0.653 | 0.653 |
| CXCL12 | CCL2 | 9606.ENSP00000379140 | 9606.ENSP00000225831 | 0 | 0 | 0.649 | 0.649 |
| CCL16 | CCL2 | 9606.ENSP00000293275 | 9606.ENSP00000225831 | 0 | 0 | 0.606 | 0.606 |
| CXCL12 | CCL7 | 9606.ENSP00000379140 | 9606.ENSP00000367832 | 0 | 0 | 0.566 | 0.566 |
| CCL7 | CCL23 | 9606.ENSP00000367832 | 9606.ENSP00000293280 | 0 | 0 | 0.564 | 0.564 |
| CCL23 | CCL2 | 9606.ENSP00000293280 | 9606.ENSP00000225831 | 0 | 0 | 0.496 | 0.496 |
| CCL23 | CCL16 | 9606.ENSP00000293280 | 9606.ENSP00000293275 | 0 | 0 | 0.471 | 0.471 |
